# Enhanced Outcrossing, Directional Selection and Transgressive Segregation Drive Evolution of Novel Phenotypes in Hybrid Swarms of the Dutch Elm Disease Pathogen *Ophiostoma novo-ulmi*

**DOI:** 10.3390/jof7060452

**Published:** 2021-06-06

**Authors:** Clive Brasier, Selma Franceschini, Jack Forster, Susan Kirk

**Affiliations:** Forest Research, Alice Holt Lodge, Farnham, Surrey GU10 4LH, UK; selmafranceschini@alice.it (S.F.); jack.forster@forestresearch.gov.uk (J.F.); susanannkirk@gmail.com (S.K.)

**Keywords:** hybridisation, pandemic, invasive species, fitness traits, perithecia, mating type, vegetative compatibility, pathogenicity

## Abstract

In the 1970s, clones of the two subspecies of *Ophiostoma novo-ulmi*, subsp. *americana* (SSAM) and subsp. *novo-ulmi* (SSNU) began to overlap in Europe, resulting in hybrid swarms. By 1983–1986, hybrids with high, SSAM-like growth and pathogenic fitness comprised ~75% of popula-tions at Limburg, Netherlands and Orvieto, Italy. We resampled these populations in 2008 to examine trends in hybrid fitness traits. Since preliminary sampling in 1979–1980, *MAT-1* locus frequency had increased from ~0% to ~32% at Orvieto and 5% to ~43% at Limburg, and vegeta-tive incompatibility type frequency had changed from near clonal to extremely diverse at both sites. This represents an enormous increase in outcrossing and recombination potential, due in part to selective acquisition (under virus pressure) of *MAT-1* and *vic* loci from the resident *O. ulmi* and in part to SSAM × SSNU hybridisation. Overt virus infection in the 2008 samples was low (~4%), diagnostic SSAM and SSNU *cu* and *col1* loci were recombinant, and no isolates exhib-ited a parental SSAM or SSNU colony pattern. At both sites, mean growth rate and mean patho-genicity to 3–5 m clonal elm were high SSAM-like, indicating sustained directional selection for these characters, though at Orvieto growth rate was slower. The once frequent SSNU-specific *up-mut* colony dimorphism was largely eliminated at both sites. Perithecia formed by Limburg isolates were mainly an extreme, long-necked SSNU-like form, consistent with transgressive segregation resulting from mismatch of SSAM and SSNU developmental loci. Orvieto isolates produced more parental-like perithecia, suggesting the extreme phenotypes may have been se-lected against. The novel phenotypes in the swarms are remodelling *O. novo-ulmi* in Europe. Locally adapted genotypes may emerge.

## 1. Introduction

Human mediated introductions of plant pathogens are resulting in novel interspecific hybridisation events, in part because previously allopatric species are less likely to have strong co-evolved genetic barriers to hybridisation [1,2,3,4,5]. Such events have the potential to generate considerable variation in fitness traits, and therefore rapid adaptive evolution, including speciation. Well documented examples occur among both the true fungi and the Oomycetes [6,7,8,9,10,11]. Particularly prominent are multiple hybridisation events among the vascular wilt pathogens that cause Dutch elm disease, with outcomes that range from hybrid swarms to introgressive hybridisation [3].

**Figure 1:**
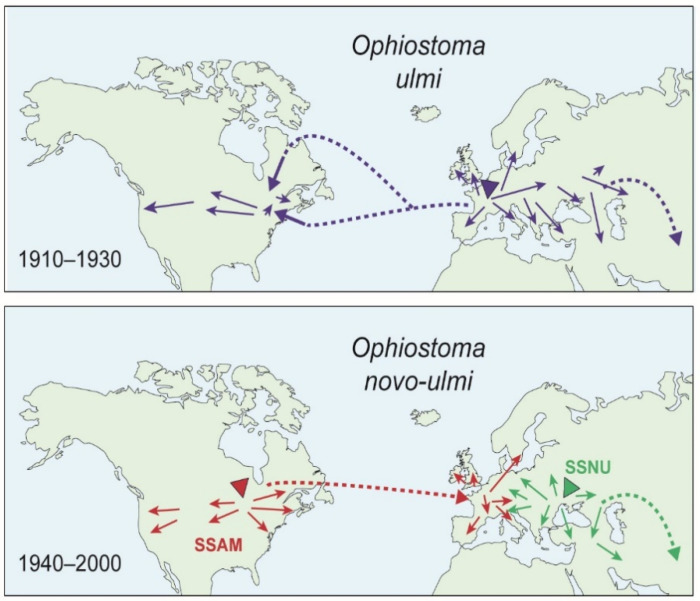
Introduction and spread of *Ophiostoma ulmi* and the *O. novo-ulmi* subspecies. Large arrowheads, primary introduction events. Small arrows, subsequent migrations. Dashed arrows, secondary importation events. Above, spread of *O. ulmi* (blue) ca. 1910–1940. Below, spread of *O. novo-ulmi* subsp. *americana* (SSAM, red) and subsp. *novo-ulmi* (SSNU, green) ca. 1940–2000. Note: (1), *O. novo-ulmi* spread into the areas previously occupied by *O. ulmi*. (2), SSAM spread from North America to western Europe in ca. 1960s. (3), SSAM and SSNU began to overlap in western Europe from ca. 1970s. Adapted from [16]. Based on characterisation of >6500 samples from the authors’ surveys [24].

There have been two highly destructive pandemics of Dutch elm disease (DED) since 1900, each caused by different *Ophiostoma* species, amounting to one of the biggest ecological disasters of the 20th Century. The first pandemic, caused by *Ophiostoma ulmi*, began in western Europe around 1910 and spread rapidly to eastern Europe and Central Asia and, in a separate introduction, to North America (Figure 1) [12]. In the mid-1900s the more aggressive *O. novo-ulmi* spread across the same regions (Figure 1), rapidly replacing *O. ulmi* [13,14] and killing most mature North American and European elms [15,16,17]. Over time it was shown that *O. ulmi and O. novo-ulmi* were adaptively very different [13,18], anciently divergent [19,20,21] and probably originated in different regions of eastern Asia [15,22], with Japan the likely origin of *O. ulmi* [23].

*Ophiostoma novo-ulmi* is polytypic, spreading mainly as two phylogenetic subspecies, subsp. *novo-ulmi* (SSNU) and subsp. *americana* (SSAM) (previously known as the Eurasian and North American races [24,25,26]. SSNU was probably first introduced into the Moscow-Romania-Caucusus area around 1940 from where it migrated westwards, reaching parts of coastal western Europe by ca. 1970s (Figure 1, green). SSAM probably appeared first in the Indiana-southern Great Lakes area of North America, again in the 1940s, and spread across the USA and to Canada (Figure 1, red). Elements of the SSAM population were subsequently introduced into the UK and neighbouring parts of western Europe from the mid-1950s to the1970s [16,27,28,29].

**Figure 2:**
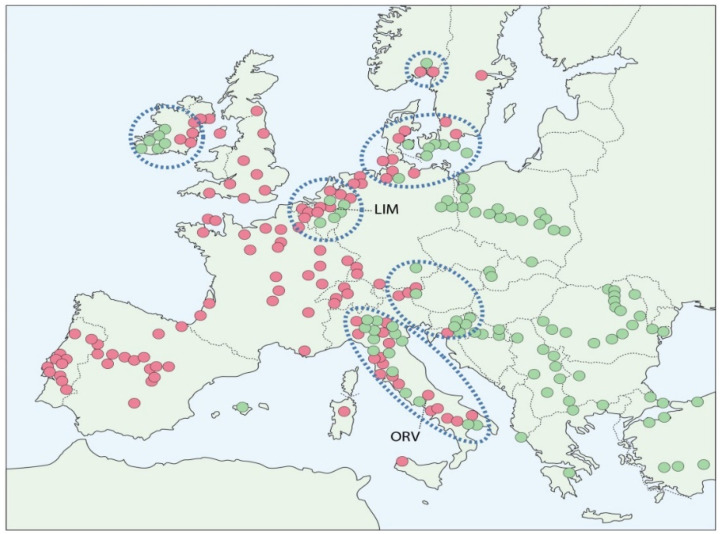
Known distribution of *Ophiostoma novo-ulmi* SSAM (red) and SSNU (green) in Europe in 1990, based on >2500 samples. Dashed blue circles, early subspecies overlap zones. ORV and LIM, locations of the Orvieto (Italy) and south Limburg (Netherlands) hybrid populations sampled in 1979–1980 and 1983–1986 and resampled in 2008. Adapted from [24], based on characterisation of >2500 samples from the authors surveys.

These sequences of introduction and migration led to three major episodes of overlap, contact and hybridisation, initially between the resident *O. ulmi* and introduced *O. novo-ulmi*, and subsequently between the *O. novo-ulmi* subspecies (Figure 1 and Figure 2). Competition between parent taxa and emerging hybrids is direct because they occupy essentially the same set of niches, represented by the pathogen’s four ecological phases [13], including a long saprotrophic phase in diseased elm bark associated with the breeding galleries of the beetle vectors. This phase results in close intermingling of different pathogen genets and, in overlap zones, of the pathogen species and subspecies [13,30,31]. Perithecia (the sexual stage) are formed during the saprotrophic phase, with fertilisation between genets aided by microarthropods [32,33]. These factors, plus the fact that *O. ulmi*, SSAM and SSNU are all obligatorily outcrossing and share a similar sexual compatibility system involving *MAT-1* and *MAT 2* types [13,34] (previously known as A and B mating-types), increased the probability of hybridisation.

SSAM and SSNU spread at epidemic fronts in Europe and North America, mainly as a few dominant, probably highly fitted, vegetative compatibility (vc) clones, subsequently confirmed to be clonal molecular lineages [29,35,36,37,38]. However, the evolutionary development of the clones in Europe and North America has been very different. The multi-locus vc systems of *O. novo-ulmi* and *O. ulmi* are central to their ecology, population structure and breeding behaviour, promoting outcrossing between ‘unlike’ vc types whilst restricting hyphal fusion and virus spread between them [13,34,39,40]. In Europe, where the frontal SSAM and SSNU clones were typically *MAT-2* [35], debilitating RNA viruses spread freely in the clones [3,35,39,41,42,43,44,45]. However, within only a few years the ‘missing’ *MAT-1* type appeared at the fronts, enabling sexual recombination; the frontal clones rapidly diversified into multiple vc types [28,35,46] and virus frequency declined [3,35,42]. During this process, transient *O. ulmi* × *O. novo-ulmi* hybrids of low fitness were also detected [47,48], and further research showed that the sudden increase in vc diversity resulted from selective acquisition and fixation, probably under the high virus pressure of *vic* loci and the *MAT-1* locus from *O. ulmi* [46,49], i.e., introgressive hybridization, the hybrids apparently acting as a genetic bridge [3,49,50,51]. *O. ulmi* DNA sequences [19,37] and some other adaptive *O. ulmi* genes were also acquired by *O. novo-ulmi*, including those controlling pathogenicity [52], ceratoulmin [53] and growth-temperature responses [54,55].

Similar *O. ulmi* > *O. novo-ulmi* introgression probably occurred in North America (Figure 1), because North American SSAM isolates also carry the *O. ulmi MAT-1* locus [46] and mitochondrial recombinants have been detected [20,37]. However, in contrast to Europe, two vc clones, the AMSG and EUSG, have remained dominant over decades, comprising ~60% of the North American SSAM population; this despite frequent sexual recombination between them generating novel vc and molecular genotypes [29,36,55,56]. The AMSG and EUSG (plus another, AM2SG, on the east coast), and their recombinants, show similar growth and pathogenic fitness. The clones are thought to have survived because, in North America, virus pressure is low [29], *O. ulmi* is near clonal in North America (in contrast to Europe) [40], there is greater competitiveness in the saprotrophic phase [29], and because American elms are more susceptible to infection [29]. When SSAM was introduced to western Europe from North America (Figure 1) it spread mainly as the EUSG, *MAT-2* type [35], though at least two other SSAM vc types also spread, the ‘Tewkesbury-Guadalajarra clone’ in western England and central Spain and the ‘Avila-Penaranda clone’ in central Spain [37,38]. The highly prevalent EUSG was subsequently introduced to New Zealand where, in the absence of both *O. ulmi* and the RNA viruses, its clonal structure remained largely unchanged [42,57].

A third major hybridisation event, and the main object of this paper, began in western and central Europe when the two subspecies of *O. novo-ulmi* began to overlap. Surveys in the 1970s–1980s revealed a predominantly westerly distribution of SSAM and easterly distribution of SSNU, reflecting their introduction and migration pathways [24,25] (Figure 1 and Figure 2). Their spread also resulted in numerous overlap-zones (Figure 2), some of which, such as those in Belgium, the Netherlands and southern Scandinavia, probably occurred through gradual migration, while others such as those in south-west Ireland, Italy and southern Norway, probably resulted from long distance ‘jumps’ due to importation of diseased elm logs or packaging material [24,25].

The two *O. novo-ulmi* subspecies differ in molecular phylogenetic markers and mtDNA size, e.g., [19,20,55,58,59,60,61,62,63]. Most importantly, they exhibit considerable phenotypic differences. Each subspecies has a different wild-type colony pattern; SSNU isolates exhibit a unique dimorphism between the striate-petaloid wild type colony form and a slow appressed *up-mut* form, and SSNU isolates are on average less pathogenic to elms and are slower growing [13,25]. SSAM forms perithecia with larger bowls but short necks, and SSNU forms perithecia with smaller bowls and longer necks [24]. They also differ in their level of ceratoulmin (cell surface hydrophobin) production [64].

The potential for subspecies hybridisation in nature is high. Only a partial, unidirectional fertility barrier is operated by SSNU against SSAM [3,13,25,34], and progenies of laboratory SSAM × SSNU F1 crosses show largely additive variation for growth rate and pathogenicity [13]. In 1979–1980, the first SSNU × SSAM hybrids with recombinant phenotypes and fertility responses were observed in samples from the Netherlands (Figure 2), and unrestricted subspecies hybridization was predicted [3,13,65]. It was soon clear that SSAM × SSNU hybrids were emerging across Europe [38,61,62,65,66,67,68,69]. To examine the frequency and properties of the emerging hybrids, samples were collected in 1983 and 1986 at two early subspecies overlap populations with different climates: south Limburg, the Netherlands, and Orvieto, Italy [68]. Based on fertility responses, colony dimorphism and molecular polymorphisms, it was shown that hybrids highly recombinant for parental molecular markers already comprised at least 78% of the Limburg and 72% of the Orvieto population. The Limburg hybrid component was shown to be just as pathogenic as the Limburg SSAM component and significantly more pathogenic than the Limburg SSNU component, indicating elimination of some SSNU-like traits by selection. Samples from a putatively ‘pure SSNU’ population in northern Poland in 1980 exhibited early, low level introgression of SSAM DNA [68] (cf. also [61]). The study therefore confirmed rapid emergence of subspecies hybrids with high growth and pathogenic fitness in overlap zones and showed that complex hybrid swarms were forming.

Examples of ‘natural’ hybrid swarms in fungi are extremely rare: a *Heterobasidion* swarm in Italy [8,9] is the only other clear example we are aware of. In the current, post epidemic period, Dutch elm disease remains one of Europe’s most damaging tree diseases [70]. The direction of the ongoing SSAM × SSNU hybridisation is therefore relevant both to the understanding of fungal hybridisation processes and to the future of native European elms. Because natural selection acts on the phenotype, a critical question is whether a common, novel phenotype of *O. novo-ulmi* will emerge from the swarms under selection or whether differently adapted phenotypes will emerge in different locations. To investigate the trends, we resampled the Limburg and Orvieto sites in 2008, after many cycles of recombination and selection since the previous 1983–1986 resamples [68]. Characters examined included perithecial architecture, growth rate and pathogenicity, colony patterns, *up-mut* colony dimorphism and diagnostic *cu* and *col-1* loci. Because both *MAT-1* frequency and vc diversity influence recombination potential in *O. novo-ulmi* [13,34] and are a product both of *O. ulmi* > *O. novo-ulmi* introgression and SSAM × SSNU hybridisation, *MAT-1* frequency and vc diversity were also investigated.

## 2. Materials and Methods

### 2.1. Comparative Site Conditions

Samples were collected across ca. 420 km^2^ of south Limburg, the Netherlands, centred on Valkenburg, and 180 km^2^ around the city of Orvieto, Umbria, Italy in September and October 2008. South Limburg has a temperate-maritime climate of average July temperature, 21 °C, and rainfall, 103mm. Orvieto is Mediterranean, with average July temperature, 31 °C, and rainfall, 19 mm. Both were typical post epidemic *Ulmus minor* sites, with thickets or hedgerows of recruitment saplings recurrently infected by *O. novo-ulmi* (see [70]). In south Limburg, these were mainly 8–20 cm diam. at 1.5 m height with good inner bark (phloem) development and relatively lush foliage. Around Orvieto, they were mainly 5–12 cm diameter at 1.5 m, with correspondingly thinner, dryer looking inner bark and more ‘scruffy’ stressed-looking foliage. At Limburg, fresh crown infections were often associated with extensive foliar wilt and xylem discolouration. At Orvieto, external and internal symptoms were more cryptic, consistent with slower dieback. Vectors were not investigated. However, at Limburg, by analogy with post-epidemic maritime southern Britain [70], they were likely to be mainly the smaller *S. multistriatus*, together with the larger and much more effective vector *S. scolytus*. At Orvieto, they were likely to be *S. multistriatus* plus the very small and even less effective *S. kirschi* and *S. pygmeus* [71,72].

### 2.2. Isolation Methods and Isolates Used in Tests

Isolations were made from 1–2 cm diameter diseased elm twigs taken from fresh infections on individual diseased trees using a selective antibiotic medium at 20–22 °C [68,73]. Details of 82 *O. novo-ulmi* isolates collected in 2008 at the Limburg and Orvieto sites and their use in different tests are shown in Table S1. The *O. novo-ulmi* isolates used as controls in *col-1* and *cu* locus analyses were: SSAM, isolates W2 (UK), RDT-38 (UK), H351 (Belgium) and H363 (Ireland); SSNU, isolates AST-27 (Iran), AST29 (Iran), H327 (Slovakia) and H413A (Germany). Hybrid isolates from the Limburg 1986 population sample used in comparative pathogenicity studies with 2008 Limburg and Orvieto isolates were: H718, H721, H735, H745, H761, H773, H784, H789 and H804 (see [68]).

### 2.3. Media and Culture Storage

Elm sapwood agar (ESA) and 2% Oxoid malt extract agar (OMEA) were prepared as described previously [72]. For Tchernoff’s liquid culture medium, see [73,74]. ESA and OMEA were poured at 15 mL/plate. Short-term stock cultures were maintained on OMEA at 20 °C and sub cultured at two-week intervals. Long-term stock cultures were maintained on OMEA slopes at −20 °C and as a glycerol and spore mixture under liquid nitrogen. For single-spore cultures, a ~1 mm^3^ piece from a colony on OMEA was blended in 10 mL of sterile water, and 0.1 mL of the resulting spore suspension was spread on an OMEA plate and incubated for 18–24 h at 20 °C; germlings were then transferred to a fresh OMEA plate using a dissecting microscope and a fine tungsten-wire needle.

### 2.4. Sexual Compatibility Types

In *O. novo-ulmi*, the sexual compatibility (or mating type) system is independent of the vc system [13,34]. The sexual compatibility type, *MAT-1 or MAT-2* (previously A and B-types), of an isolate was determined by the presence or absence of perithecia when conidia of the ‘unknown’ isolate, scraped from the surface of a 10 day old colony on OMEA, were applied to a 1 cm^2^ patch on a mature colony of *MAT-1* tester isolate MM2-1 on ESA [73]. The sexual compatibility types of individual Limburg and Orvieto isolates are shown in Table S1.

### 2.5. Vegetative Compatibility Type Tests

In the *O. novo-ulmi* vc system, the multiple *vic-w* loci are epistatic to the multiple *vic-n* loci [13,34,56]. Eleven isolates within the Limburg sample and eleven within the Orvieto sample were paired in all possible combinations on 9 cm ESA plates. Inocula of ~3 mm diam, cut from the edges of 3–5 day-old colonies on OMEA, were placed centrally 10 mm apart, and the plates were incubated at 20 °C in darkness for 21 days. They were scored for the vc reaction patterns common to SSAM and SSNU [13,34,36]. Isolates of the same vc type (isogenic for all *vic* loci) intermingle at the colony junction line, with no synnemetal (asexual fruiting structure) formation, producing a compatible or C-reaction. Isolates sharing the same *vic-w l*oci but having different *vic-n* loci are incompatible and produce a dense narrow mycelial barrage ~3–5 mm wide at the junction line, usually accompanied by synnematal formation within a few mm of the barrage, termed a ‘narrow’ or n-reaction. Isolates having different *vic-w l*oci produce a mycelial barrage of ~10–20 mm width at the junction line and synnematal formation 10–30 mm beyond the barrage, termed a ‘wide’ or w-reaction. Pairings were also scored for perithecia, demonstrating sexual compatibility, using a dissecting microscope and incident light.

### 2.6. Growth Rate, Colony Morphology and Pathogenicity Tests

Combined growth rate and colony morphology tests were carried out on OMEA, with two replicate plates per isolate. Two colony diameters were measured at right angles after two and seven days incubation at 20 °C, followed by ten days further incubation in diffuse light [73]. Two additional colony morphology tests were carried out under the same conditions to allow maximum expression of *up-mut* colony dimorphism characteristic of SSNU [26] and to detect unstable ‘amoeboid’ colonies characteristic of overt RNA virus infection [3,39,41,42].

Pathogenicity tests were carried out at the Forest Research trial ground, Sleaford, Hampshire on blocks of five-year-old, ~3–3.5 m tall, moderately susceptible clonal English elm (*Ulmus procera* clone SR4, a form of *U. minor*) and five-year-old, ~4–5 m tall, moderately resistant clonal Commelin elm (*U*. × ‘Commelin’, clone 274) at 1 m spacing, pruned to a more lenticular shape to aid disease assessment (Figures S1–S4). Isolates were derived from single spores, allotted a random code number, inoculated to a 10 mL universal bottle containing 5 mL of Tchernoff’s liquid medium, and shaken at room temperature for 3 days to induce the yeast stage of the pathogen [73,74]. Two drops of spore suspension (ca. 1 × 10^5^ spores mL^−1^) were inoculated into the current annual ring of each tree one fifth (*U. procera*) or half-way (*U*. × ‘Commelin’) down the crown using a scalpel and a hypodermic syringe. Each experiment was randomized overall, with four replicate trees per isolate.

Disease as percentage defoliation of the crown was assessed after 12 weeks by three independent assessors. Mean percentages (calculated from logits) were calculated across the three assessments for each tree species, with separate pairwise comparisons of population-level differences and within-population isolate differences assessed separately using generalised linear models with logit link function and quasibinomial errors (to account for overdispersion). Analysis of deviance (likelihood ratio χ^2^ tests) were used to test for significant differences between populations and isolates within populations.

### 2.7. Production of Perithecia for Morphological Comparisons

Pairings of *MAT-1* and *MAT-2* Limburg or Orvieto isolates were set up simultaneously on sterilised split elm twigs [24,74]. Healthy *U. procera* twigs ~1 cm diameter were peeled, cut to 6 cm lengths, split in half and autoclaved under water. Isolates were grown for 3 days on OMEA at 20 °C. For each isolate pair, a sterile filter paper was placed in the bottom of a 9 cm petri dish and moistened with sterile water; three sterile split twigs were added by resting each twig section, flat surface downwards, on two 15 mm diam × 3 mm-high sterilised plastic rings. Four alternating equidistant MAT-1 and MAT-2, ca. 2 mm^3^ inoculum plugs were placed on each twig section, two of them close to the ends. The plate was then sealed with Parafilm^®^ (Bemis Inc., Oshkosh, WI, USA; PM992) and incubated in darkness for 21 days at 20 °C.

For each pairing, 20 mature perithecia were removed under 16× magnification by an independent technician using a fine tungsten-needle loop, transferred to a smear of lactic acid on a microscope well slide, covered with a rectangular coverslip and the neck lengths and bowl widths of 15 random perithecia per pairing measured. Isolates in Limburg pairings comprised (*MAT-1* × *MAT-2*): pairing A, NL20 × NL52; B, NL51B × NL21; C, NL45 × NL53; D, NL1 × NL16; E, NL40 × NL27; F, NL23 × NL57; G, NL10 × NL4; H, NL36 × NL51; I, NL32 × NL28; J, NL58 × NL35. Those in Orvieto pairings comprised (MAT-1 × MAT-2): pairing K, I352 × I350; L, I366 × I341; M, I344 × I346; N, I351 × I364; O, I362 × I356; P, I366 × I368; Q, I345 × I343. Owing to a scarcity of *MAT-1* isolates among the Orvieto sample, one random isolate, I366, was duplicated in pairings L and P.

Perithecial dimensions were analysed using [75]. An initial data clean was conducted to remove atypical morphologies. Neck length to bowl width ratios and neck length data were transformed to square-roots to meet the assumptions of normality. Neck length to bowl width ratios were analysed separately for Limburg and Orvieto isolates using a generalised least squares model ([76], gls function), allowing within-pairing variances to differ. After fitting the model, the mean of each pairing was compared against the pooled mean of all other pairings (excluding the most extreme minimum and maximum values) to identify pairings falling outside the expected morphology of the two sub-species, i.e., as statistically atypical. Normal distribution assumptions were then rechecked via visual inspection of a set of residual diagnostics (histogram of residual values, fitted values versus residual plot, normal QQ plot and half normal QQ plot). On this basis, Limburg pairings D, G and H and Orvieto pairings L and Q were excluded from the comparisons of the Limburg and Orvieto means.

Using the cleaned sub-species data sets, neck lengths, bowl widths and NL: BW ratios of the pairings were analysed using mixed effects models ([76], lme function), including sub-species as a fixed effect and pairings as a random effect with sub-species specific variances. Analysis of variance, based on the model results, was used to determine significant differences by sub-species and between pairings within subspecies, and Tukey’s HSD test for multiple comparisons was used post-hoc to assess differences between means.

### 2.8. DNA Extraction and RFLP Analysis of Cu and Col-1 Loci

DNA extractions were performed on freeze-dried mycelium obtained from cultures grown over sterilized cellophane [77]. For each isolate, 100 mg of mycelium was extracted with Dneasy Plant mini Kit (Qiagen^®^, Hilden, Germany) following the company’s instructions. The genomic DNA was then amplified using forward and reverse primers of *cu* and *col-1* locus, respectively [78,79].

Cerato-ulmin is a single locus cell surface hydrophobin present on *O. novo-ulmi* mycelium and spores [80]. The cerato-ulmin hydrophobin (*cu*) gene sequences of SSAM and SSNU differ at a substitution site [78]. The endonuclease *Hph1* cuts the *cu* locus of SSNU at position 250 twice and SSMA only once, resulting in three polymorphic banding patterns in SSNU and two in SSAM [61]. The *O. novo-ulmi col-1* locus is involved in differentiation of the hyphal and yeast growth phases [79]. The *col-1* loci of SSAM and SSNU differ at six nucleotide sites in a 482 bp region, and the restriction enzyme Bfa1 results in three polymorphic bands in SSAM and two in SSNU [61].

Isolates selected for the RFLP analyses comprised fourteen ‘unknown’ isolates taken at random from each of the Orvieto and Limburg sites (Table S1) and four SSAM and four SSNU reference isolates (listed above). Each PCR reaction contained 1 μL of undiluted DNA sample, 1 unit of Go Taq, 5 μL of PCR Green Buffer (Promega^®^, Madison, WI, USA), 2 μL of 2nm dNTPs mixture (Promega^®^), 1 μL of 10 μL primer and 14.8 μL of molecular grade water (Sigma^®^, St. Louis, MO, USA). The PCR cycle for the *cu* locus consisted of a denaturation step at 94 °C for 4 min, followed by 30 cycles of 0.15 min at 94 °C, 1 min at 68 °C and 2 min at 72 °C, and a final extension step of 5 min at 72 °C. The PCR program for the *col-1* locus consisted of a denaturation step at 94 °C for 2 min, followed by 35 cycles of 0.3 min at 94 °C, 0.3 min at 58 °C and 1 min at 72 °C, and a final extension step of 10 min at 72 °C. Five μL aliquots of each sample were loaded in a 1.2% agarose gel to verify amplicon presence, quantity and size.

Amplicons of the *cu* locus were digested with *Hph1* (Fermentas^®^, London, UK) and *col-1* fragments with Bfa1 (New England Biolabs, Ipswich, MA, USA). RFLP reactions were performed in 15 μL of final volume. Each digestion mixture consisted of 5 μL of PCR product, 2.5 U of enzyme, 1.5 μL of 10× buffer and 8.25 μL of nuclease free water. Tubes were incubated at 37 °C for 5 h. Five μl of RFLP samples were visualised on 3% agarose (Sigma^®^, St. Louis, MO, USA) gel stained with 5 μL/100 mL GelRed (Biotium, CA, USA) alongside a 100 Bp (Promega^®^, Madison, WI, USA) and a Low Range Gene ruler (Fermentas ^®^, London, UK) DNA ladder. Gel images were acquired after 90 min at 100 V.

## 3. Results

### 3.1. Increase in MAT-1 Frequency at the Limburg and Orvieto Sites

The frequency of *MAT-1* at the Limburg and Orvieto hybrid sites has been investigated since 1979–1980, the early subspecies overlap phase. At Limburg in 1980, 14 sample isolates were identified as SSAM and 8 as SSNU, of which 21 were *MAT-2* and only one was *MAT-1* (Table 1). By 1983, when ~79% of the isolates were SSAM × SSNU hybrids, *MAT-1* frequency had increased to ~35%. In the present 2008 sample, the hybrid swarm phase, with hybrid frequency assumed to be ~100%, 42.8% of the sample was *MAT-1*, i.e., closer to but short of a 1:1 ratio for *MAT-1*:*MAT-2*.

Similarly at Orvieto in 1979 (Table 1), seven isolates were identified as SSAM and eight as SSNU, and all were *MAT-2*. By 1986, when at least ~72% of Orvieto isolates were hybrids, 10.5% were *MAT-1*s. In 2008, *MAT-1* frequency had advanced to 28% but was still well short of a 1:1 ratio (*p* = 0.0432). The difference in frequency of *MAT-1*s at Orvieto and Limburg in 2008 was not significant (LR χ^2^ = 1.66, *p* = 0.198)), probably due to the sample sizes. Nonetheless, the data suggest a slower increase in MAT-1 frequency at Orvieto than at Limburg.

### 3.2. Vegetative Compatibility Type Diversity in the 2008 Limburg and Orvieto Populations

**Figure 3:**
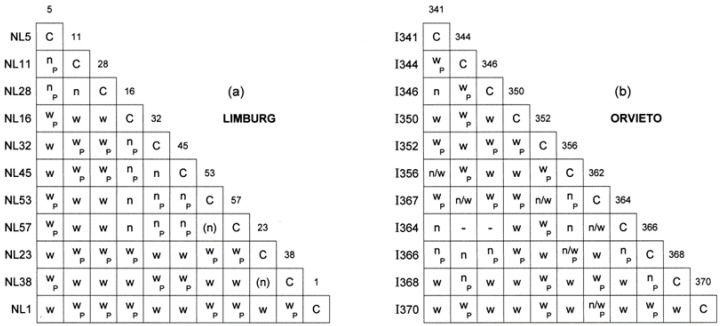
Vegetative compatibility reactions between paired *O. novo-ulmi* subspecies hybrid isolates from the Limburg (**a**) and Orvieto (**b**) populations. C, compatible reaction; W, incompatible ‘wide’ barrage reaction; n, incompatible ‘narrow’ barrage reaction; P, perithecia formed (sexually compatible pairing); n/w, unclear whether an n- or a w-reaction; (n), weaker n-reaction; -, no data.

Diversity in vegetative compatibility (vc) types at the Limburg and Orvieto sites is expected to be a product of introgression of *vic* loci from *O. ulmi*, the *vic* loci of the overlapping SSAM and SSNU clones, and *vic* locus recombination resulting from the introgression of the *MAT-1*s. To examine vc diversity in 2008, the hybrid swarm stage, eleven Limburg and eleven Orvieto isolates were paired all × all (Figure 3).

All self × self (control) pairings resulted in a compatible (C-) reaction. In the Limburg set (Figure 3a), all 55 self × non-self pairings gave incompatible (w- or n) reaction, i.e., every isolate was of a unique vc type. Isolates NL5, 11 and 28 gave an n-reaction together but w-reactions with the other isolates, indicating they share the same *vic-w* allele but have different *vic-n* alleles. Isolates NL16, 32, 45, 53 and 57 also gave n-reactions together and therefore shared a different *w*-allele. Isolate NL1 gave a w-reaction against all the other isolates and therefore had a unique *w*-allele. Overall, there were four *w*-alleles among the 11 isolates tested. The number of *n*-alleles was at least five, as indicated by the five isolates all giving n-reactions against each other.

In the Orvieto set (Figure 3b) all 53 self × non-self pairings also produced incompatible reactions. However, in 11 pairings whether the reaction was an n- or a w-reaction could not be discriminated from the structure of the mycelial barrage and associated synemmatal formation, an issue not previously encountered in *O. novo-ulmi* vc tests. These reactions were scored n/w. This limited the overall interpretation of the interaction matrix. However, as isolates I350, I352 and I370 gave only, or almost only, w-reactions against all other isolates, including each other, it is again likely that at least four different *vic-w* alleles were present among the 11 isolates.

As all self × non-self pairing combinations were incompatible, the probability of any one random pair of isolates at the Limburg or Orvieto sites being vegetatively incompatible was close to 100%.

### 3.3. Growth Rates, Colony Patterns, Up-Mut Dimorphism and Virus Infection in the 2008 Limburg and Orvieto Hybrids

When the mean radial growth rates of the 55 Limburg and 26 Orvieto 2008 isolates were compared (Table 2), the Orvieto isolates grew on average significantly more slowly, at mean 3.24 mm/day ^−1^ (range 2.3–4.1), than the Limburg isolates, at mean 3.54 mm /day ^−1^ (range 2.9–4.5) (F_1.78_ = 10.3, *p* = 0.0019).

The colony patterns of the isolates were highly variable, and it was not possible to assign them to either the SSAM or SSNU colony type. While the SSNU-specific *up-mut* colony dimorphism was present at 35–50% of the 1979–1986 Limburg and Orvieto samples (Table 3), in the 2008 samples none of the Orvieto isolates and only two of the Limburg isolates (3.6%) exhibited the dimorphism. None of the Orvieto isolates exhibited the unstable ‘amoeboid’ colonies characteristic of overt virus infection, but three Limburg isolates (NL43, NL44 and NL48) did so, indicating low level virus infection (~5.3%) at the site.

### 3.4. Comparative Pathogenicity of the 2008 Limburg and Orvieto Hybrids

The pathogenicity of subsets of the 2008 Limburg and Orvieto samples and of the 1986 Limburg hybrid component were compared on English and Commelin elm, with fifteen isolates in each sample set (Table 2; Figures S1–S4). On both elm types, the 1986 Limburg hybrid sample caused marginally but not significantly greater mean defoliation than the 2008 Limburg sample. Similarly, the mean defoliation caused by the 2008 Orvieto sample, although slightly higher than that caused by the Limburg 2008 sample, was not significantly different from it. Nor was it significantly different from the Limburg 1986 mean.

### 3.5. Perithecial Form in the Limburg and Orvieto Hybrids

**Figure 4:**
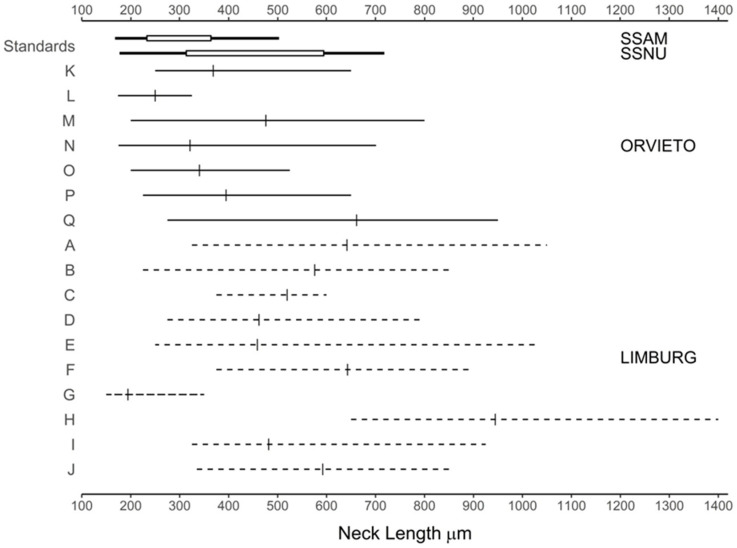
Comparative perithecial neck lengths of the Orvieto and Limburg hybrids and of the parental *O. novo-ulmi* subspecies. Above, SSAM and SSNU standards: published neck length ranges (thicker horizontal lines) and range of means (narrow horizontal boxes) for the two subspecies [24], based on pairings of 14 SSAM and 16 SSNU isolates from across Eurasia and North America. Middle and bottom, means and ranges for the seven pairings, K to G, within the Orvieto hybrid population and the ten pairings, A to J, within the Limburg hybrid population. Solid (Orvieto) and dashed (Limburg) horizontal lines, sample ranges. Short vertical bars, sample means. Details of isolate pairings in Section 2.7.

**Figure 5:**
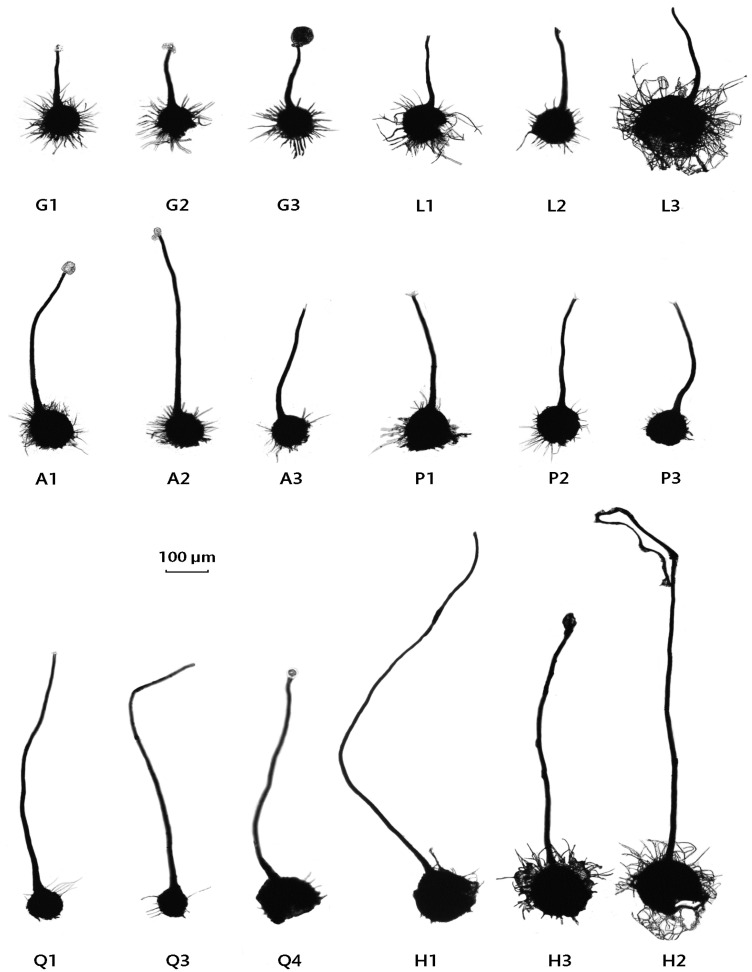
Perithecia of Orvieto and Limburg hybrids. Top row, random perithecia of short-necked, more SSAM-like Limburg and Orvieto pairings, G and L, respectively; Middle row, random perithecia of more SSNU-like Limburg and Orvieto pairings, A and P, respectively; bottom row, random perithecia of extreme long necked Orvieto and Limburg pairings, Q and H, respectively. Scale bar = 100 μm. Some perithecial bowls are adorned with setae. Dark droplets at the tips of some necks are extruded ascospore masses. Parings: G, isolates NL10 × NL4; L, I366 × I368; A, Nl20 × NL52; P, I366 × I341; Q, I345 × I343; H, NL36 × NL51. See also Figure 4 and Figure 6.

**Figure 6:**
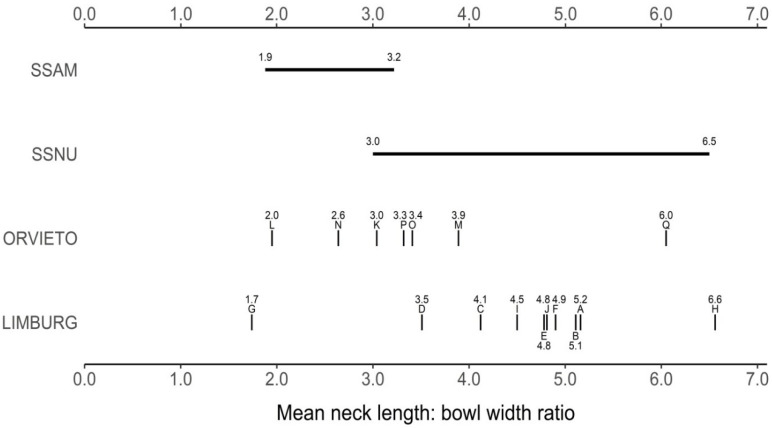
Mean perithecial neck length: bowl width ratios (NL: BW) of the Orvieto and Limburg hybrids and the parental *O. novo-ulmi* subspecies. Above, horizontal bars, SSAM, SSNU standards: published range of NL: BW means for the two subspecies [24]. Below, short vertical bars, NL: BW means of the seven Orvieto pairings, L to Q (middle row), and the ten Limburg pairings, A to J (bottom row). Details of isolate pairings in Section 2.7.

The perithecial dimensions of the 2008 hybrids were investigated in ten within-Limburg (A to J) and seven within-Orvieto (K to Q) *MAT-1* × *MAT-2* pairings and compared to those previously recorded for SSAM and SSNU (Table 4; Figure 4, Figure 5 and Figure 6). All the perithecia contained ascospores. In the Orvieto set, the mean neck length of pairing L conformed to the SSAM type, those of two pairings, N and O, lay between SSAM and SSNU, and three, K, M and P, lay at the lower end of the SSNU type (Figure 4 and Figure 5; Table 4). The neck length of pairing Q, however, considerably exceeded that previously recorded for SSNU, both in terms of its mean (675 μm ) and maximum (950 μm ) neck length.

The Limburg set exhibited from unusually short to unusually long perithecial necks (Figure 4 and Figure 5, Table 4). Pairing G had necks with a mean length of 197 μm, below the lowest previously recorded mean for SSAM of 233 μm. The means of six pairings, B, C, D, E, I and J, lay within the previously recorded range of means of SSNU, but in five of these the maximum neck length of 800–1050 μm considerably exceeded the maximum recorded neck length for SSNU of 718 μm. In pairings A and F, both the mean and the maximum neck lengths exceeded, and in pairing H (at mean 950 μm and maximum 1400 μm) greatly exceeded, the previously recorded metrics for SSNU.

The overall mean neck length of the Limburg pairings, at 535 μm, was also considerably greater than that of the Orvieto pairings at 393.0 μm (Table 4), but the means were not significant owing to the large variance, both within pairings and within the sites. However, when statistically atypical pairings D, G and H for the Limburg set and L and Q for the Orvieto set were omitted, resulting in adjusted means of 556.8 μm and 378.3 μm, respectively, and reduced within site variance, the Limburg and Orvieto sets were significantly different (Table 4). In this adjusted analysis, the mean neck length of the Limburg set was also significantly different from that of the parental SSAM type, but not from SSNU, whereas the mean of the adjusted Orvieto set was significantly different from SSNU but not from SSAM (Table 4). When the parental data (SSAM and SSNU) were pooled, the Limburg mean, but not the Orvieto mean, was significantly different from the combined parent mean (Table 4). The mean perithecial bowl widths of the Limburg and Orvieto sets were not significantly different, whether or not pairings G, L and N were omitted (Table 4).

The neck length (NL): bowl width (BW) ratios, which reflect overall perithecial form, were also informative (Figure 6 and Table 4). In the Limburg set, the mean ratio of pairing G (1.74) again fell below the lowest mean previously recorded for SSAM (1.88). Otherwise, despite tending to produce longer necks than SSNU, the mean ratios of the other nine Limburg pairings all lay within, or close to, the recorded range of means for SSNU. The adjusted overall mean ratio of seven Limburg pairings (i.e., excluding statistically atypical pairings D, G and H), at 4.66, was also not significantly, different from the overall recorded mean for SSNU (Table 4). Therefore, while the perithecial necks of the adjusted Limburg set tended to be longer than those of SSNU, the perithecia retained SSNU-like proportions, i.e., the perithecial bowls tended to be commensurately wider.

In the Orvieto set the overall mean NL: BW ratio, at 3.32, was at the low end of the recorded SSNU range of means and considerably below the Limburg unadjusted mean at 4.35 (Table 4 and Figure 6). Indeed, although Orvieto pairing Q, at ratio 6.05, fell near the upper range of SSNU means (Figure 6), the means of the other six pairings fell within the recorded range of means of SSAM or SSNU. The Orvieto means were therefore skewed towards more SSAM-like proportions. Based again on the adjusted data (omitting atypical Orvieto pairings L and Q), the mean Orvieto NL: BW ratio of 3.19 was still not significantly different from that of SSAM (2.52); whereas the mean of the adjusted Limburg set, at 4.66, was significantly greater than that of both the Orvieto and the SSAM means (Table 4). When the parental (SSAM and SSNU) data were pooled, the Limburg mean, but not the Orvieto mean, was significantly different from the combined parent mean (Table 4).

### 3.6. Frequency of the SSAM and SSNU Cu and Col-1 Genes

The frequency of the subspecies diagnostic SSAM and SSNU cerato-ulmin (*cu*) and *col-1* loci in the 2008 hybrid samples was examined in subsets of 14 Limburg and 14 Orvieto isolates, together with five SSAM and five SSNU controls. For both loci, the controls gave the expected parental banding patterns.

With the *cu* locus (Table 5), in the Limburg set, twelve isolates (86%) exhibited the SSNU pattern and only two isolates (14%) the SSAM pattern, whereas, in the Orvieto set, eight (57%) exhibited the SSNU pattern and six (43%) the SSAM pattern (LR χ^2^ = 2.90, *p* = 0.089). Although this might indicate selection against the SSAM *cu* allele at the Limburg site, it is more likely to reflect the small sample size.

With *col-1* (Table 5), nine of the Limburg isolates (64%) exhibited the SSAM and five (36%) the SSNU pattern, and eight Orvieto isolates (57%) the SSAM and six (43%) the SSNU pattern (LR χ^2^ = 0.15, *p* = 0.699), suggesting the subspecies *col-1* alleles are selectively neutral at both sites.

Despite the small sample sizes and the low frequency of the SSAM *cu* allele in the Limburg subset, three of the four possible *cu/col-1* allele combinations were represented in the Limburg subset and all four in the Orvieto subset (Table 5). Additionally, with the combined Limburg and Orvieto data, the difference between the number of parental and recombinant genotypes (17, 11; Table 5) was not significant (*p* = 0.345). The *cu and col-1* loci therefore appear to be relatively freely recombining among the hybrids. When the sexual compatibility (*MAT-1* and *MAT-2*) types of the isolates were included (Table 5), seven of the eight possible *cu/col1/MAT* combinations were represented in the combined data, indicating relatively free recombination also between *cu, col-1* and *MAT*.

## 4. Discussion

Previously we showed rapid emergence of hybrids between the two introduced *O. novo-ulmi* subspecies at the Limburg and Orvieto early overlap sites. By 1983–1986, the frequency of *O. novo-ulmi* subspecies hybrids at the Limburg and Orvieto sites had already reached 70–80% [68]. In this study, we re-examined the same populations after ~25 years, investigating changes in key fitness characters that can be broadly divided into those related to introgression from the resident *O. ulmi* and those influenced mainly by subspecies hybridisation, summarised in Table 6.

### 4.1. Trends in Characters Associated with O. ulmi > O. novo-ulmi Introgression

*MAT-1* frequency and vc diversity are critical to the evolution and behaviour of introduced *O. novo-ulmi* populations. Selective acquisition of the *O. ulmi MAT-1* locus by *MAT-2 O. novo-ulmi* clones at epidemic fronts, whether involving ‘pure’ SSAM, ‘pure’ SSNU, or SSAM / SSNU overlap populations, has apparently occurred repeatedly across Europe and North America since the 1940s [3,28,35,46]. In Europe at least, this process was apparently driven by the spread of viruses in the frontal clones. Together with a simultaneous acquisition of novel *vic* alleles from *O. ulmi*, it led to the appearance of novel vc types and a marked decline in overt virus infection [2,3,28,35,38,42,45,46].

We show here that at Orvieto and Limburg the frequency of *MAT-1* increased from 0–5% in 1979–1980 to 28–43% in 2008. Because isolates carrying an introgressed *MAT-1* locus tend to be more fecund (as female) than *MAT-2*s, producing many more protoperithecia, the influence of *MAT-1*s on outcrossing potential is greater than their simple numerical frequency [13,34]. Hence, a frequency of 43% *MAT-1*s could even be optimal for recombination. Certainly, the steady increase in *MAT-1*s will have enhanced recombination initially between the parent subspecies and subsequently among the hybrid genotypes.

By analogy with ‘pure’ SSAM and ‘pure’ SSNU populations spreading at European epidemic fronts the Limburg and Orvieto, early overlap populations (Figure 2) are likely to have comprised a single SSAM vc clone and a single SSNU vc clone, most probably the predominant and widespread EUSG and EANSG clones, respectively [28,35]. We show here that, by 2008, as in the ‘pure’ SSAM or ‘pure’ SSNU populations, the Limburg and Orvieto hybrid populations had become highly diverse, the 11 isolates tested per site being unique vc types. This change will have resulted (a) from the acquisition of novel *vic* alleles from *O. ulmi*; (b) from the different *vic-w* and *vic-n* alleles contributed by each subspecies; and (c) from enhanced outcrossing due to the increasing frequency of *MAT-1*s. Furthermore, because *MAT-1* × *MAT-2* pairings giving incompatible wide (w-) vc reactions are considerably more fecund than those giving incompatible narrow (n-) reactions [13,34], any increase in *w*-alleles will have further enhanced outcrossing potential. We observed a minimum of four *w*-alleles at both the Limburg and the Orvieto sites, making the probability of a w- as opposed to an n-reaction between random *MAT-1* × *MAT-2* pairings around 74.5% at the Limburg site and 66% at the Orvieto site. Previously undescribed ‘n/w –reactions’ occurred in some Orvieto pairings (Figure 3b). These could be *O. ulmi*-like vc reactions (cf. [40]) due to a functional mismatch between *O. novo-ulmi vic* loci and introgressed *O. ulmi vic* loci.

By reducing pathogen growth rate and conidial viability, the RNA viruses (d-factors) that spread in the frontal SSAM and SSNU clones [41,43,44,45,77] can significantly suppress the pathogen’s ability to infect elms [39,81,82]. However, following the increase in vc diversity, overt virus infection in the ‘pure’ SSAM and ‘pure’ SSNU populations fell to low levels [28,35,42]. This has probably also been the case at subspecies overlap sites. In the present study, none of the 26 Orvieto isolates and three of the 55 Limburg isolates were overtly virus infected (~5% overall). Virus spread will have been suppressed not only by the high vc diversity but also by the enhanced ascospore formation resulting from increased sexual reproduction. Nonetheless, it appears that virus infection has not been fully suppressed. This may reflect the continued prevalence of narrow (n-) vc-reactions (Figure 3), which are less effective than w-reactions in supressing virus transmission [13,34,39,42].

In terms of changes in *MAT-1* frequency, vc diversity and overt virus infection, the Limburg and Orvieto hybrid populations appear to have followed the same trends as ‘pure’ SSAM or SSNU populations in Europe.

### 4.2. Trends in Characters Associated with SSAM × SSNU Hybridisation

By 1983–1986 the hybrid components at Limburg and Orvieto already exhibited a high level of recombination between SSAM and SSNU molecular markers [68]. In the present study, the combinations of SSAM and SSNU-specific *cu* and *col-1* loci [61,78] also demonstrated a high level of recombination among the hybrids. Although both loci are located on chromosome IV [79,83], there was no indication of significant linkage between *cu* and *col-1*, or between these loci and *MAT*. The persistence of the SSAM and SSNU alleles of these loci at both sites suggests they are largely selectively neutral. A comparatively low frequency of the SSAM *cu* gene at Limburg (14.2%, Table 5) probably reflects the small sample size rather than selection.

Several of the major character differences between SSAM and SSNU are continuously variable phenotypic traits. Because many genes often act in concert in such characters, they can be complex to interpret, yet critical to understanding whether a character is responding to selection [84]. One such character not previously examined in SSAM × SSNU hybrids but markedly different in each subspecies is perithecial morphology. SSAM forms perithecia with large bowls and shorter necks, SSNU the reverse [24]. We have shown here a divergence in perithecial form at the Limburg and Orvieto sites and greater variation than that previously recorded for both subspecies combined at the Limburg site. Thus, one Limburg pairing produced perithecia with an average neck length considerably below that recorded for SSAM. The other nine pairings produced perithecia with mean and maximum neck lengths often in excess of that recorded for SSNU, and in one pairing they were much greater than previously recorded. Despite this, the mean neck length: bowl width ratios of these nine pairings fell within the range expected for SSNU. These perithecia were therefore also considerably more robust overall but still in proportion to an SSNU-like architecture.

In contrast, the perithecial necks of the Orvieto pairings were mostly of a short SSNU type or short to regular SSAM type. In consequence, the overall mean neck length of the adjusted Orvieto sample, at 378.3 μm, was significantly lower than that of the Limburg sample, at 556.8 μm. However, one Orvieto pairing also produced perithecia with unusually long necks, i.e., similar to many Limburg pairings.

The marked differences in the perithecia produced by the Limburg and Orvieto isolates raises the question of whether selection is involved. It is difficult to envisage what biological advantage might be conferred by the extreme, long-necked perithecia produced by the Limburg sample. Indeed they might be more vulnerable to bending and therefore to ascospore secretion failure. In the absence of studies on the genetic control of perithecial architecture in SSAM and SSNU, however, it is only possible to speculate. Given that, even by 1983–1986, high recombination levels for parental SSAM and SSNU markers were recorded at both sites, it is reasonable to assume that high levels of recombination are also likely to have occurred between loci governing developmental processes. Given also that the mean neck length and mean NL: BW ratio of the Limburg perithecia differ significantly from the combined parental (SSAM and SSNU) means, we favour the view that the extreme phenotypes at Limburg are due to instability resulting from mismatches between SSAM and SSNU loci involved in perithecial development, i.e., that they are transgressive segregation phenotypes (cf. [85]; and Goldschmidt’s [86] ‘Hopeful Monsters’). On this basis, it is likely that similar extreme phenotypes also appeared at Orvieto, but have been largely eliminated by selection.

Growth fitness is critical to most aspects of *O. novo-ulmi* development, from colonisation of beetle feeding wounds and breeding galleries to reproductive competence, territorial antagonism between genets and re-infestation of the vectors. Pathogenicity is also critical to competitive ability during colonization of the bark and feeding wounds as well as the vascular wilt phase in elm xylem [13,30,31]. In the laboratory, SSAM × SSNU crosses of both characters showed high additivity [26,87]. A previous study on the Limburg population in 1986 showed that selection favoured hybrid genotypes with high SSAM-like mean pathogenicity and growth rate. In the present study, the 2008 Limburg hybrids were just as highly pathogenic as the 1986 Limburg hybrids, and the 2008 Orvieto hybrids were equally pathogenic. The Orvieto 2008 hybrids were also fast growing on average, though significantly slower growing than the 2008 Limburg hybrids. It appears, therefore, that selection has favoured higher growth rate and pathogenicity at both sites, i.e., these characters appear to be under sustained directional selection. This is in agreement with evidence from Britain that, in the current post epidemic period, selection for high growth rate and high aggressiveness has probably increased since the main epidemic period [70].

This trend is paralleled by the change in the frequency of the *up-mut* colony dimorphism observed at the two sites. This striking and unusual dimorphism is characteristic of the SSNU parent and contributes to its overall slower average growth rate and lower pathogenicity [13,26]. In the 1983–1986 samples, the *up-mut* dimorphism was present in 35% of the Limburg and 50% of the Orvieto isolates (combined SSNU and hybrid components [68]. In the present tests, however, only one of the 81 hybrid isolates exhibited the dimorphism, indicating the trait was close to being eliminated. Its function is unknown, but the loss is almost certainly another consequence of selection favouring faster growth and higher pathogenicity. The distinctive parental SSAM or SSNU colony patterns have also been lost; in the present tests, the hybrid isolates were highly variable and none could be assigned to a parental type. Hence, continued recombination among the hybrids has resulted in an array of novel, largely unclassifiable colony patterns.

Overall, therefore, there are many similarities in the main character trends among the 2008 Limburg and Orvieto hybrids (Table 6), but there are also notable differences. For example, at Orvieto, the differences included a lower *MAT-1* frequency, a slower average growth rate and more conservative perithecial architecture. Many factors are likely to result in differential selection at the sites, including local climate, elm genotype, climate × elm effects, vector species and microbial antagonists [13]. Thus, at Limburg, the maritime climate and the thicker and moister inner bark of the *U. minor* should be more favourable to pathogen and vector development, and the likely main vectors at Limburg, *S. scolytus* and *S. multistriatus*, more effective at pathogen transmission. It could be argued, therefore, that more intense environmental stresses at Orvieto may have contributed to the differences observed between the sites. However, such generalisations are further complicated by the influence of pleiotropy. For example, some genes involved in *O. novo-ulmi* growth rate are probably also involved in pathogenicity [88].

The evidence that selection in the post epidemic period has favoured faster growth and higher pathogenic aggressiveness in the hybrids does raise the question of whether selection imposed by a large scale deployment of highly disease resistant elms, cf. [89,90] could result in a further increase in aggressiveness in *O. novo-ulmi*, i.e., an increase sufficient to result in higher disease levels in previously resistant elm stock. Because disease levels in the DED ‘system’ involve a range of biologically and environmentally influenced critical thresholds [13,30,70], any attempts to predict them are problematic. Nonetheless, accepting that genetic control of pathogenicity in *O. novo-ulmi* is multigenic and largely additive, even under current, probably stringent post-epidemic conditions, the potential for higher mean aggressiveness may already be near the maximum [70]. Thus, given the ecologically complex, multi-phase life cycle of *O. novo-ulmi*, any response to selection for increased pathogenicity at the population level is likely to be balanced against the need to maintain fitness across a range of other fitness parameters, including competitive ability in the saprotrophic phase and reproductive efficiency [13,31]. Achieving maximal fitness for one parameter is likely to exert a cost on maintaining fitness for another. A further marked increase in *O. novo-ulmi* aggressiveness would therefore appear to be unlikely.

Every introduced pathogen is an uncontrolled, open-ended experiment in evolution [91], and hybridisation only adds to the uncertainty of any outcome. Both the *O. ulmi* × *O. novo-ulm* and *O. novo-ulmi* SSAM × SSNU hybridisation processes in Europe have been highly dynamic, involving migrating fronts, and both have proceeded extremely rapidly, but there the main comparisons end. Progenies of *O. ulmi* × *O. novo-ulmi* crosses exhibit female sterility and very strong negative interactions for growth rate, pathogenicity and bark colonising ability, i.e., they show extreme transgressive segregation [13,50,92,93]. In nature, although *O. ulmi* × *O. novo-ulmi* hybridisation occurred repeatedly at epidemic fronts, the hybrids were readily outcompeted by *O. novo-ulmi* and the main outcome, while evolutionarily very significant, was selective introgression and the near extinction of *O. ulmi*. In contrast, not only has SSAM × SSNU recombination occurred freely but, as shown here, the hybrids and their descendants are well fitted for competition with the parent subspecies and are replacing them. Further, a range of novel *O. novo-ulmi* phenotypes have emerged: in the hybrid zones, *O. novo-ulmi* is being substantially remodelled in Europe. The possibility that locally adapted phenotypes, or ecotypes, of *O. novo-ulmi* will emerge from the swarms is demonstrated by the different trends at Limburg and Orvieto.

## 5. Conclusions

Between 1979 and 2008, as a result of both introgression from *Ophiostoma ulmi* and hybridisation between the *Ophiostoma novo-ulmi* subspecies, SSAM and SSNU, *MAT-1* frequency at two hybrid zones in Limburg, Netherlands and Orvieto, Italy has risen from near zero to 30–40% and vc diversity from near-clonal to very high, a dramatic increase in outcrossing potential. Overt virus infection at the sites is at a low level, probably as a consequence of the high vc diversity and increased ascospore formation. Enhanced outcrossing has also enhanced recombination of loci governing SSAM and SSNU characteristics.

There are many similarities and some differences in how selection is moulding the hybrid swarms at Limburg and Orvieto. At both sites, continued directional selection has apparently favoured genotypes with high, SSAM-like growth rate and high pathogenic aggressiveness, although at Orvieto, mean growth rate is slower. Directional selection has probably also led to loss of the SSNU-specific *up-mut* colony dimorphism at the sites. Extreme perithecial phenotypes formed by the Limburg sample probably reflect mismatches between SSAM and SSNU developmental loci. At Orvieto, these extreme perithecial forms may have been largely eliminated. Novel phenotype combinations in the swarms are remodelling *O. novo-ulmi* in Europe. Locally adapted genotypes may emerge.

## Figures and Tables

**Table 1:** Change in frequency of the *MAT-1* sexual compatibility type at the Limburg and Orvieto sites.

Sample and Date	Limburg			Orvieto		
	No. of Isolates	% Hybrid	% *MAT-1*	No. of Isolates	% Hybrid	% *MAT-1*
Early subspecies overlap phase, 1979–1980	22	4.5 ^1^	4.5 ^3^	15	~0 ^2^	0
Advanced hybrid phase, 1983–1986	96	>79 ^4^	35.4 ^3^	108	>72 ^4^	10.5 ^3^
Hybrid swarm phase, 2008 ^5^	56	~100	42.8	25	~100	28.0

^1^ Data from [65]. ^2^ Estimate: only subspecies and *MAT* investigated in 1979. ^3^ Previously unpublished data of authors. ^4^ Data from [68]. ^5^ Contrast, % *MAT-1*, 2008 Limburg vs 2008 Orvieto: *p* > 0.05 (χ^2^).

**Table 2:** Comparative growth rate and pathogenicity of Limburg and Orvieto samples.

Sample ^1,2^	Mean Growth Rate and SE mm /Day^−1^	Mean Pathogenicity (% Defoliation) and SE	
		English Elm SR4	Commelin Elm 270
2008 Orvieto	3.24 ± 0.08 (55) ^1^	42.4 ± 4.5 (15)	17.1 ± 2.2 (15)
2008 Limburg	3.54 ± 0.05 (26)	38.2 ± 4.5 (15)	12.0 ± 1.9 (15)
1986 Limburg	nt ^3^	41.9 ± 4.5 (15)	18.0 ± 2.2 (15)

^1^ Number of isolates tested in parentheses. ^2^ Contrasts: Growth rate, 2008 Orvieto vs. 2008 Limburg, significantly different (F_1.78_ = 10.3, *p* = 0.0019). Pathogenicity English elm, 2008 Orvieto vs. 2008 Limburg, and 2008 Limburg vs. 1986 Limburg, both *p* > 0.05 (LR χ^2^). Pathogenicity Commelin elm, 2008 Orvieto vs. 2008 Limburg, and 2008 Limburg vs. 1986 Limburg, both *p* > 0.05 (LR χ^2^). ^3^ nt, not tested. There were no significant differences between isolates within samples on English elm. For Commelin elm, global models indicated significant differences between the 2008 Orvieto (LR Chi-Sq = 20.7, *p* = 0.014), 2008 Limburg (LR Chi-Sq = 17.4, *p* = 0.042) and 1986 Limburg hybrid (LR Chi-Sq = 20.7, *p* = 0.014) isolates.

**Table 3:** Change in frequency of the *up-mut* colony dimorphism at the Limburg and Orvieto sites.

Sample and Date	Limburg		Orvieto	
	No. of Isolates	% *Up-Mut*	No. of Isolates	% *Up-Mut*
Early subsp. overlap phase 1979–1980	22	36.4 ^1^	15	53.3 ^1^
Advanced hybrid phase 1983–1986	107	52.3 ^2^	92	34.8 ^2^
Hybrid swarm phase 2008	56	0	25	0

^1^ Previously unpublished data of authors. ^2^ Data from [68].

**Table 4:** Perithecial dimensions of within-Limburg and within-Orvieto pairings and of the parent subspecies.

Groups	No. of Pairings	Neck Length (NL) μm			Bowl Width (BW) μm			NL: BW Ratio		
		Mean	Contrast		Mean	Contrast		Mean	Contrast	
			1 ^3^	2 ^4^		1	2 ^4^		1	2 ^4^
*All data*										
Orvieto	7	393.1 ± 57.4	NS	NS	120.0 ± 4.5	NS	NS	3.32 ± 0.40	NS	NS
Limburg	10	535.2 ± 56.3			123.2 ± 3.8			4.35 ± 0.38		
*Adjusted* ^1^										
Orvieto	5	378.3 ± 26.0	a	a	119.7 ± 5.0	a	a	3.19 ± 0.31	ab	ab
Limburg	9	556.8 ± 28.4	b	b	120.0 ± 4.2	a	a	4.66 ± 0.32	c	b
*Subspecies* ^2^										
SSAM	10	294.0 ± 129.7	a		115.5 ± 12.5	a		2.52 ± 0.34	a	
SSNU	10	444.0 ± 162.8	b		103.4 ± 12.4	a		4.30 ± 0.42	bc	
Subsp. mean	20	366.6 ± 45.03		a	109.4 ± 8.1		a	3.41 ± 0.24		a

^1^ Omitting statistically atypical pairings L and Q (Orvieto) and D, G and H (Limburg). ^2^ Based on raw data in [24] for seven within-SSAM pairings of fourteen isolates from North America and Europe and eight within-SSNU pairings of sixteen isolates across Eurasia, under the same conditions. ^3^ Tukey’s HSD test significance, location means versus individual subspecies means. ^4^ Tukey’s HSD test significance, location means versus pooled subspecies mean. Rows within a column not sharing a letter were significantly different at a *p* < 0.05. NS, not significant.

**Table 5:** Frequency of SSAM and SSNU *cu* and *col-1* loci and *MAT* types in subsamples of the Limburg and Orvieto isolates ^1^.

Locus Combination		No. of Isolates		Total	of Which	
*cu* Locus	*col-1* Locus	Limburg	Orvieto		*MAT-1*	*MAT-2*
SSNU	SSNU	5	5	10	5	5
SSAM	SSAM	2	5	7	5	2
SSNU	SSAM	7	3	10	4	6
SSAM	SSNU	0	1	1	0	1

^1^ Genotypes of individual isolates shown in Table S1.

**Table 6:** Main hybrid trends at Limburg and Orvieto since the early subspecies overlap phase, 1979–1980 ^1^.

Character	Trend	Inferred Evolutionary Process
**Substantially *O. ulmi* > *O. novo-ulmi* Introgression-Related**		
*MAT-1* frequency	Increase from near zero to 28% (**Orvieto**) and 43% (**Limburg**)	Directional selection (under virus pressure)
Diversity of vc types	Change from near clonal to very high diversity	Directional selection (under virus pressure)
Overt virus infection	Greatly reduced	Suppression by high vc diversity and ascospore production
**Subspecies Hybridisation-Related**		
Growth rate	Fast, SSAM-like; slower at **Orvieto**	Directional selection
*Up-mut* dimorphism	Loss	Directional selection
Colony pattern	Loss of original subspecies patterns; highly variable	Uncertain
Pathogenicity	High, SSAM-like	Directional selection
Perithecial neck length	**Limburg**: extreme phenotypes; mean length significantly above combined subspecies mean	Transgressive segregation
	**Orvieto**: from SSAM-like to shorter necked SSNU-like, mean not significantly different from combined subspecies mean	Transgressive segregation followed by stabilising selection
Perithecial neck length: bowl width ratio	**Limburg**: mean ratio significantly higher than combined subspecies mean	Transgressive segregation
	**Orvieto**: mean not significantly different from combined subspecies mean	Transgressive segregation followed by stabilising selection
SSSAM and SSNU *cu* loci	Co-occurrence	Neutral
SSSAM and SSNU *col-1* loci	Co-occurrence	Neutral

^1^ Based on current data (see Table 4) and data in [68]. Boldface discriminates where there are important trend differences between the Limburg and Orvieto sample sites and saves splitting the column.

## Data Availability

Raw data are stored at Forest Research, Farnham, UK.

## Supplementary Materials

The following are available online at https://www.mdpi.com/article/10.3390/jof7060452/s1, Table S1: Details of *O. novo-ulmi* isolates collected at the Limburg and Orvieto sites in 2008 including *MAT* types, *cu* locus and *col-1* locus polymorphisms, and use of the isolates in the different tests, Figure S1: Plot of ~3–3.5 m clonal English elm (*U. procera*) four weeks after inoculation, showing early symptoms (wilting and browning) at the tops of the crowns, Figure S2: Plot of ~3–3.5 m clonal English elm (*U. procera*) four weeks after inoculation, showing early symptoms (wilting and browning) at the tops of the crowns, Figure S3: Plot of ~4–5 m clonal Commelin elm (*U.* × Commelin) four weeks after inoculation, showing early symptoms (wilting and browning) at the tops of the crowns, Figure S4: Plot of ~4–5 m clonal Commelin elm (*U.* × Commelin) four weeks after inoculation.

## References

[1] Brasier C.M. (1995). Episodic selection as a force in fungal microevolution with special reference to clonal speciation and hybrid introgression. Can. J. Bot..

[2] Brasier C.M. (2000). Rise of the hybrid fungi. Nature.

[3] Brasier C.M. (2001). Rapid evolution of introduced plant pathogens via interspecific hybridization. Bioscience.

[4] Depotter R.L., Seidl R.F., Wood T.A., Thomma B.P.H.J. (2016). Interspecific hybrization impacts host range and pathogenicity of filamentous microbes. Curr. Op. Microbiol..

[5] Stukenbrock E.H. (2016). The Role of Hybridization in the evolution and emergence of new fungal plant pathogens. Phytopathology.

[6] Brasier C.M., Cooke D., Duncan J.M. (1999). Origins of a new *Phytophthora* pathogen through interspecific hybridisation. Proc. Natl. Acad. Sci. USA.

[7] Newcombe G., Stirling B., McDonald S., Bradshaw H.D. (2000). *Melampsora* × *columbiana,* a natural hybrid of *M. medusae and M. ocidentalis*. Mycol. Res..

[8] Gonthier P., Nicolotti G., Linzer R., Guglielmo F., Garbelotto M. (2007). Invasion of European pine stands by a North American forest pathogen and its hybridization with a native interfertile taxon. Mol. Ecol..

[9] Fabiano S., Garbelotto M., Giordano L., Gonthier P. (2021). Genic introgression from an invasive exotic fungal forest pathogen increases the establishment of potential odf a sibling native pathogen. NeoBiota.

[10] Stukenbrock E.H., Christiansen F.B., Hansen T.T., Dutheil J.Y. (2012). Fusion of two divergent fungal individuals led to the recent emergence of a unique widespread pathogen species. Proc. Natl. Acad. Sci. USA.

[11] Husson C., Aguayo J., Revellin C., Frey P., Marcais B. (2015). Evidence for homoploid speciation in *Phytophthora alni* supports taxonomic reclassification in this species complex. Fungal Genet. Biol..

[12] Gibbs J.N. (1978). Intercontinental epidemiology of Dutch elm disease. Ann. Rev. Phytopathol..

[13] Brasier C.M. (1986). The population biology of Dutch elm disease: Its principal features and some implications for other host-pathogen systems. Adv. Plant Pathol..

[14] Brasier C.M., Mitchell A.G., Kirk S.A. (1990). Decline of the non-aggressive subgrouop at current epidemic fronts. Report on Forest Research.

[15] Brasier C.M. (1990). China and the origins of Dutch elm disease: An appraisal. Plant Pathol..

[16] Brasier C.M., Dunne C.P. (2000). Intercontinental spread and continuing evolution of the Dutch elm disease pathogens. The Elms: Breeding, Conservation and Disease Management.

[17] Brasier C.M., Buck K.W. (2002). Rapid evolutionary changes in a globally invading pathogen, the causal agent of Dutch elm disease. Biol. Invasions.

[18] Brasier C.M. (1991). *Ophiostoma novo-ulmi* sp. nov., causative agent of the current Dutch elm disease pandemic. Mycopathologia.

[19] Bates M.R., Buck K.W., Brasier C.M. (1993). Molecular relationships between *Ophiostoma ulmi* and the EAN and NAN races of *O. novo-ulmi* determined by restriction fragment length polymorphisms of nuclear DNA. Mycol. Res..

[20] Bates M.R., Buck K.W., Brasier C.M. (1993). Molecular relationships of the mitochondrial DNA of *Ophiostoma ulmi* and the NAN and EAN races of *O. novo-ulmi* determined by restriction fragment length polymorphisms. Mycol. Res..

[21] Pipe N.D., Brasier C.M., Buck K.W. (2000). Evolutionary relationship of the Dutch elm disease fungus *Ophiostoma novo-ulmi* to other *Ophiostoma* species investigated by RFLP analysis of the rDNA region. J. Phytopathol..

[22] Brasier C.M., Mehrotra M.D. (1995). *Ophiostoma himal-ulmi* sp. nov., a new species of Dutch elm disease fungus endemic to the Himalayas. Mycol. Res..

[23] Masuya M., Brasier C., Ichihara Y., Kubono T., Kanzaki N. (2009). First report of the Dutch elm disease pathogens *Ophiostoma ulmi* and *O. novo-ulmi* in Japan. Plant Pathol. New Dis. Reps..

[24] Brasier C.M., Kirk S.A. (2001). Designation of the EAN and NAN races of *Ophiostoma novo-ulmi* as subspecies. Mycol. Res..

[25] Brasier C.M. (1979). Dual origin of recent Dutch elm disease outbreaks in Europe. Nature.

[26] Brasier C.M. (1986). A comparison of pathogenicity and cultural characteristics in the EAN and NAN aggressive sub-groups of *Ophiostoma ulmi*. Trans. Brit. Mycol. Soc..

[27] Brasier C.M., Gibbs J.N. (1973). Origin of the Dutch elm disease epidemic in Britain. Nature.

[28] Brasier C.M., Kirk S.A. (1991). Rapid changes in *O. novo-ulmi* population structure at current epidemic fronts. Report on Forest Research.

[29] Brasier C.M., Kirk S.A. (2000). Survival of clones of NAN *Ophiostoma novo-ulmi* around its probable centre of appearance in North America. Mycol. Res..

[30] Webber J.F., Brasier C.M., Anderson J.M., Rayner A.D.M., Walton D. (1984). The transmission of Dutch elm disease: A study of the processes involved. Invertebrate-Microbial Interactions.

[31] Webber J.F., Brasier C.M., Mitchell A.G., Pegg G.F., Ayres P.G. (1988). The role of the saprophytic phase in Dutch elm disease. Plant Infecting Fungi.

[32] Lea J., Brasier C.M. (1983). A fruiting succession in *Ceratocystis ulmi* and its role in Dutch elm disease. Trans. Brit. Mycol. Soc..

[33] Brasier C.M. (1978). Mites and reproduction in *Ceratocystis ulmi* and other fungi. Trans. Brit. Mycol. Soc..

[34] Brasier C.M., Jennings D., Rayner A.D.M. (1984). Inter-mycelial recognition systems in *Ceratocystis ulmi*: Their physiological properties and ecological importance. The Ecology and Physiology of the Fungal Mycelium.

[35] Brasier C.M. (1988). Rapid changes in genetic structure of epidemic populations of *Ophiostoma ulmi*. Nature.

[36] Brasier C.M. (1996). Low genetic diversity of the *Ophiostoma novo-ulmi* population in North America. Mycologia.

[37] Bates M.R. (1990). DNA Polymorphism in the Dutch Elm Disease Fungus *Ophiostoma ulmi*. Ph.D. Thesis.

[38] Brasier C.M., Buck K.W., Paoletti M., Crawford L., Kirk S.A. (2004). Molecular analysis of evolutionary changes in populations of *Ophiostoma novo-ulmi*. Investig. Agrar. Sist. Recur. For..

[39] Brasier C.M., Buck K.W. (1986). The d-factor in *Ceratocystis ulmi*: Its biological characteristics and implications for Dutch elm disease. Fungal Virology.

[40] Mitchell A.G., Brasier C.M. (1994). Contrasting structure of European and North American populations of *Ophiostoma ulmi*. Mycol. Res..

[41] Brasier C.M. (1983). A cytoplasmically transmitted disease of *Ceratocystis ulmi*. Nature.

[42] Brasier C.M., Dunne C.P. (2000). Viruses as biological control agents of the Dutch elm disease pathogens. The Elms: Breeding, Conservation and Disease Management.

[43] Hong V., Dover S.L., Cole T.E., Brasier C.M., Buck K.W. (1999). Multiple mitochondrial viruses in an isolate of the Dutch elm disease fungus *Ophiostoma novo-ulmi*. Virology.

[44] Buck K.W., Brasier C.M., Tavantzis S. (2001). Viruses of the Dutch elm disease pathogens. Molecular Variability of Double Stranded RNA. Concepts and Applications in Agriculture and Forestry and Medicine.

[45] Buck K.W., Brasier C.M., Paoletti M., Crawford L., Hails R.S., Beringer J.E., Godfrey H.A. (2002). Virus transmission and gene flow between two species of Dutch elm disease fungi, *Ophiostoma ulmi* and *O.novo-ulmi*: Deleterious viruses as selective agents for gene introgression. Genes in the Environment.

[46] Paoletti M., Buck K.W., Brasier C.M. (2006). Selective acquisition of novel mating type and vegetative incompatibility genes via interspecies gene transfer in the globally invading eukaryote *Ophiostoma novo-ulmi*. Mol. Ecol..

[47] Brasier C.M., Kirk S.A., Pipe N., Buck K.W. (1998). Rare hybrids in natural populations of the Dutch elm disease pathogens *Ophiostoma ulmi* and *O. novo-ulmi*. Mycol. Res..

[48] Kirisits T., Konrad H. (2004). Dutch elm disease in Austria. For. Res. Syst..

[49] Bates M.R., Brasier C.M., Buck K.W. (1990). Dutch elm disease. Source of the rapid variation in the aggressive subgroup at epidemic fronts. Report on Forest Research.

[50] Kile G.A., Brasier C.M. (1990). Inheritance and inter-relationship of fitness characters in progeny of aggressive × non-aggressive crosses of *Ophiostoma ulmi*. Mycol. Res..

[51] Brasier C.M., Kirk S.A. (1993). Rare hybrids may be a genetic bridge between *Ophiostoma ulmi* and *O. novo-ulmi*. Report on Forest Research.

[52] Abdelali A., Brasier C.M., Bernier L. (1999). Localization of a pathogenicity gene in *Ophiostoma novo-ulmi* and evidence that it may be introgressed from *O. ulmi*. Mol. Plant Microbe Interact..

[53] Pipe N.D., Brasier C.M., Buck K.W. (2001). Two natural ceratoulmin deficient mutants of *Ophiostoma novo-ulmi*: One has an introgressed *O. ulmi cu* gene; the other has an *O. novo-ulmi cu* gene with a mutation in an intron splice concensus sequence. Mol. Plant Pathol..

[54] Et-Touil A., Dusabenyagasani M., Bouvet G.F., Brasier C.M., Bernier L. (2019). *Ophiostoma ulmi* DNA naturally introgressed into an isolate of *Ophiostoma novo-ulmi* is clustered around pathogenicity and mating type loci. Phytoprotection.

[55] Hessenauer P., Fijarczyk A., Martin H., Prunier J., Charron G., Chapuis J., Bernier L., Tanguay P., Hamelin R., Landry C. (2020). Hybridization and introgression drive genome evolution of the Dutch elm disease pathogens. Nat. Ecol. Evol..

[56] Millgroom M.G., Brasier C.M. (1997). Potential diversity of vegetative compatibility types of *Ophiostoma novo-ulmi* in North America. Mycologia.

[57] Brasier C.M., Gadgil P. (1992). Dutch elm disease outbreak in New Zealand. Report on Forest Research.

[58] Hoegger P.J., Binz T., Heiniger U. (1996). Detection of genetic variation between *Ophiostoma ulmi* and the EAN and NAN races of *O. novo-ulmi* determined by restriction fragment length polymorphisms of the nuclear DNA. Mycol. Res..

[59] Jeng R.S., Bernier L., Brasier C.M. (1988). A comparative study of cultural and electrophoretic characteristics of the Eurasian and North American races of *Ophiostoma ulmi*. Can. J. Bot..

[60] Pipe N.D., Buck K.W., Brasier C.M. (1995). Molecular relationships between *Ophiostoma ulmi* and the EAN and NAN races of *O. novo-ulmi* determined by RAPD markers. Mycol. Res..

[61] Konrad H., Kirisits T., Riegler M., Halmschlager E., Stauffer C. (2002). Genetic evidence for natural hybridisation between the Dutch elm disease pathogens *Ophiostoma novo-ulmi* ssp. *novo-ulmi* and *Ophiostoma novo-ulmi* ssp. *americana*. Plant Pathol..

[62] Santini A., Montaghi A., Vendramin G.G., Capretti P. (2005). Analysis of the Italian Dutch elm disease population. J. Phytopathol..

[63] Paoletti M., Buck K.W., Brasier C.M. (2005). Cloning and sequence analysis of the *Mat*-B (*Mat*-2) genes from the three Dutch elm disease pathogens *Ophiostoma ulmi, O. novo-ulmi* and *Ophiostoma himal-ulmi*. Mycol. Res..

[64] Brasier C.M., Takai S., Nordin J.H., Richards W.C. (1990). Differences in ceratoulmin production between EAN, NAN and non-aggressive subgroups of *Ophiostoma ulmi*. Plant Pathol..

[65] Brasier C.M. (1986). Dutch elm disease—*Ophiostoma (Ceratocystis) ulmi*. The emergence of EAN and NAN hybrids in Europe. Report on Forest Research.

[66] Dvořák M., Tomšovský M., Jankovský L., Novotný D. (2007). Contribution to identify the causal agents of Dutch elm disease in the Czech Republic. Plant Protect. Sci..

[67] Solla A., Dacasa M.C., Nasmith C., Hubbes M., Gil L. (2008). Analysis of Spanish populations of *Ophiostoma ulmi* and *O. novo-ulmi* using phenotypic characters and RAPD markers. Plant Pathol..

[68] Brasier C.M., Kirk S.A. (2010). Rapid emergence of hybrids between the two subspecies of *Ophiostoma novo-ulmi* with a high level of pathogenic fitness. Plant Pathol..

[69] Katović Z., Krstin L., Ćaleta B., Stanĉin P., Zebec M., Ćurković-Peria M. (2020). Identification and characteriosation of the causal agent of Dutch elm disease in Croatia. Eur. J. For. Res..

[70] Brasier C.M., Webber J.F. (2019). Is there evidence for post-epidemic attenuation in the Dutch elm disease pathogen *Ophiostoma novo-ulmi*?. Plant Pathol..

[71] Webber J.F. (1990). Relative effectiveness of *Scolytus scolytus*, *S. multistriatus* and *S. kirschi* as vectors of Dutch elm disease. Eur. J. For. Pathol..

[72] Favaro A., Battista A. (1993). Observations on the elm bark beetle *Scolytus pygmaeus* as a possible vector of the fungus *Ophiostoma ulmi*. Redia.

[73] Brasier C.M., Stipes R.J., Campana R.J. (1981). Laboratory investigation of *Ceratocystis ulmi*. Compendium of Elm Diseases.

[74] Tchernoff B. (1965). Methods for screening and for the rapid selection of elms for resistance to Dutch elm disease. Acta Bot. Neer..

[75] R Core Team R: A Language and Environment for Statistical Computing. R Foundation for Statistical Computing. https://www.R-project.org/-.

[76] Pinheiro J., Bates D., DebRoy S., Sarkar D. (2018). R Core Team: Linear and Nonlinear Mixed Effects Models. R Package Version 3.1-137. https://CRAN.R-project.org/package=nlme.

[77] Rogers H.J., Buck K.W., Brasier C.M., Buck K.W. (1986). The molecular nature of the d^2^ factor in *Ceratocystis ulmi*. Fungal Virology.

[78] Pipe N., Buck K.W., Brasier C.M. (1997). Comparison of cerato-ulmin (cu) gene sequences of the recently identified Himalayan Dutch elm disease fungus *Ophiostoma himal-ulmi* with those of *O. ulmi* and *O. novo-ulmi* suggests the cu gene of *O. novo-ulmi* is unlikely to have been acquired recently from *O. himal-ulmi*. Mycol. Res..

[79] Pereira V., Hintz W.E., Royer J.C., Field D.J. (2000). A gene associated with filamentous growth in *Ophiostoma novo-ulmi* has RNA-binding motifs and is similar to a yeast gene involved in RNA splicing. Curr. Genet..

[80] Takai S. (1974). Pathogenoicity and cerato-ulmin production in *Ceratocystis ulmi*. Nature.

[81] Webber J.F. (1987). Influence of the d^2^ factor on survival and infection by the Dutch elm disease pathogen *Ophiostoma ulmi*. Plant Pathol..

[82] Sutherland M.L., Brasier C.M. (1997). A comparison of thirteen d-factors as potential biological control agents of *Ophiostoma novo-ulmi*. Plant Pathol..

[83] Bowden C.G., Hintz W.E., Jeng R., Hubbes M., Horgen P.A. (1994). Isolation and characterization of the cerato-ulmin toxin gene of the Dutch elm disease pathogen, *Ophiostoma ulmi*. Curr. Genet..

[84] Brasier C.M., Worrall J. (1998). Fitness, continuous variation and selection in fungal populations: An ecological perspective. The Structure of Fungal Populations.

[85] Rieser L.H., Archer A.A., Wayne R.K. (1999). Transgressive hybridization, adaptation and speciation. Heredity.

[86] Goldschmidt R.B. (1940). The Material Basis of Evolution.

[87] Brasier C., Day P.R., Jellis G.J. (1987). Some genetical aspects of necrotophy with special reference to *Ophiostoma ulmi*. Genetics and Plant Pathogenesis.

[88] Brasier C.M., Webber J.F. (1987). Positive correlations between in vitro growth rate and pathogenesis in *Ophiostoma ulmi*. Plant Pathol..

[89] Santini A., Pecori F., Pepori A., Brookes A. (2012). ‘Morfeo’ Elm: A new variety resistant to Dutch elm disease. For. Pathol..

[90] Martín J.A., Sobrino-Plata J., Rodríguez-Calcerrada J. (2019). Breeding and scientific advances in the fight against Dutch elm disease: Will they allow the use of elms in forest restoration?. New For..

[91] Brasier C.M. (2008). The biosecurity threat to the UK and global environment from international trade in plants. Plant Pathol..

[92] Brasier C.M., Gibbs J.N. (1976). Inheritance of pathogenicity and cultural characters in *Ceratocystis ulmi*: I. Hybridisation of aggressive and non-aggressive strains. Ann. Appl. Biol..

[93] Brasier C.M. (1977). Inheritance of pathogenicity and cultural characters in *Ceratocystis ulmi*: Hybridisation of protoperithecial and non-aggressive strains. Trans. Brit. Mycol. Soc..

