# Supplementary material for: Enhanced Outcrossing, Directional Selection and Transgressive Segregation Drive Evolution of Novel Phenotypes in Hybrid Swarms of the Dutch Elm Disease Pathogen Ophiostoma novo-ulmi
Source: J Fungi (Basel). 2021 Jun 6;7(6):452. doi: 10.3390/jof7060452 (PMC8228177; doi:10.3390/jof7060452)
Supplement: Supplementary file 1 [file jof-07-00452-s001.zip › jof-1228785-SI/Supplementary files/Supplementary Figure legends.pdf]

Supplementary figure legends

Figure S1. Plot of ~ 3-3.5 m clonal English elm (*U. procera*() four weeks after inoculation, showing early symptoms (wilting and browning) at the tops of the crowns.

Figure S2. Plot of ~ 3-3.5 m clonal English elm (*U. procera*() four weeks after inoculation, showing early symptoms (wilting and browning) at the tops of the crowns.

Figure S3. Plot of ~4-5 m clonal Commelin elm (*U.* x Commelin*)* four weeks after inoculation showing early symptoms (wilting and browning) at the tops of the crowns.

Figure S4. Plot of ~4-5 m clonal Commelin elm (*U.* x Commelin*)* four weeks after inoculation.
