# Supplementary material for: Enhanced Outcrossing, Directional Selection and Transgressive Segregation Drive Evolution of Novel Phenotypes in Hybrid Swarms of the Dutch Elm Disease Pathogen Ophiostoma novo-ulmi
Source: J Fungi (Basel). 2021 Jun 6;7(6):452. doi: 10.3390/jof7060452 (PMC8228177; doi:10.3390/jof7060452)
Supplement: Supplementary file 1 [file jof-07-00452-s001.zip › jof-1228785-SI/Supplementary files/Table S1 Sampled isolates.pdf]

Table S1. Sexual compatibility types, *cu* and *col-1* alleles and experimental use of 2008 Orvieto and Limburg isolates.

Isolate no.	Sexual compatibility type	*cu* locus/ *col-1* locus [1]	Isolate used in assessment of [2]			
			Growth rate / colony morphology	Pathogenicity	Perithecial morphology	Vegetative compatibility
Orvieto						
I 341	*MAT*-2	am / nu	X	X	X	X
I 343	*MAT*-2	nu / nu	X		X	
I 344	*MAT*-1	am / am	X	X	X	X
I 345	*MAT*-1	nu / nu	X		X	
I 346	*MAT*-2	nu / am2	X	X	X	X
I 348A	*MAT*-2	am / am	X			
I 348B	*MAT*-2		X			
I 350	*MAT*-2		X	X	X	X
I 351	*MAT*-1	am / am	X		X	
I 352	*MAT*-1	am / am	X	X	X	X
I 355	*MAT*-2		X			
I 356	*MAT*-2	nu / nu	X	X	X	X
I 358	*MAT*-2		X			
I 359	*MAT*-2	nu / nu	X	X		X
I 360	*MAT*-2		X			
I 362	*MAT*-1	nu / nu	X	X	X	X
I 363	*MAT*-2		X			
I 364	*MAT*-2	nu / am	X	X	X	X
I 365	*MAT*-1		X			
I 366	*MAT*-1	nu / am	X	X	X	X
I 367	*MAT*-2		X			
I 368	*MAT*-2	am / am	X		X	X
I 369	*MAT*-2		X			
I 370A	*MAT*-2		X			X
I 370B	*MAT*-2		X			
Limburg						
NL1	*MAT*-1	nu / nu	X	X	X	X
NL2	*MAT*-2		X			
NL3	*MAT*-1		X			
NL4	*MAT*-2	nu / am	X		X	
NL5	*MAT*-1		X	X		X
NL6	*MAT*-2		X			
NL7	*MAT*-1		X			
NL9	*MAT*-2		X			
NL10	*MAT*-1		X		X	
NL11	*MAT*-2	nu / nu	X	X		X
NL12	*MAT*-2		X			
NL 13	*MAT*-2		X			
NL 14	*MAT*-2		X			
NL 15	*MAT*-2		X			
NL 16	*MAT*-2		X	X	X	X
NL 17	*MAT*-1		X			
NL 18	*MAT*-1		X			
NL 19	*MAT*-1		X			

Isolate no.	Sexual compatibility type	*cu* locus/ *col-1* locus [1]	Isolate used in assessment of [2]			
			Growth rate / colony morphology	Pathogenicity	Perithecial morphology	Vegetative compatibility
NL20	*MAT*-1	nu / nu	X		X	
NL21	*MAT*-2		X		X	
NL22	*MAT*-2		X			
NL23	*MAT*-1	nu / am	X	X	X	X
NL24	*MAT*-2		X			
NL25	*MAT*-1		X			
NL26	*MAT*-2		X			
NL27	*MAT*-2	nu / am	X		X	
NL28	*MAT*-2		X	X	X	X
NL29	*MAT*-2		X			
NL30	*MAT*-1		X			
NL31	*MAT*-1		X			
NL32	*MAT*-1	am / am	X		X	X
NL33	*MAT*-1		X			
NL34	*MAT*-1		X			
NL35	*MAT*-2	nu / nu	X		X	
NL36	*MAT*-1		X		X	
NL37	*MAT*-1		X			
NL38	*MAT*-2		X	X		X
NL 39	*MAT*-1		X			
NL40	*MAT*-1	nu / nu	X		X	
NL41	*MAT*-2		X			
NL42	*MAT*-2		X			
NL43	*MAT*-2		X			
NL44	*MAT*-2		X			
NL45	*MAT*-1	nu / am	X		X	
NL46	*MAT*-1		X			
NL 47	*MAT*-2	nu / am	X			
NL48	*MAT*-2		X	X		
NL49	*MAT*-2		X			
NL50	*MAT*-1	am / am	X			
NL51	*MAT*-2		nt [3]		X	
NL51B	*MAT*-1		X		X	
NL52	*MAT*-2		X	X	X	
NL53	*MAT*-2	nu / am	X		X	X
NL54	*MAT*-2		X			
NL55	*MAT*-1		X			
NL56	*MAT*-2		X			
NL57	*MAT*-2		X	X	X	X
NL58	*MAT*-1	nu / am	X		X	

[1] am: SSAM allele; nu: SSNU allele. [2] X: isolate used in tests. [3] nt, not tested
