# Supplementary figures and images for: Enhanced Outcrossing, Directional Selection and Transgressive Segregation Drive Evolution of Novel Phenotypes in Hybrid Swarms of the Dutch Elm Disease Pathogen Ophiostoma novo-ulmi
Source: J Fungi (Basel). 2021 Jun 6;7(6):452. doi: 10.3390/jof7060452 (PMC8228177; doi:10.3390/jof7060452)

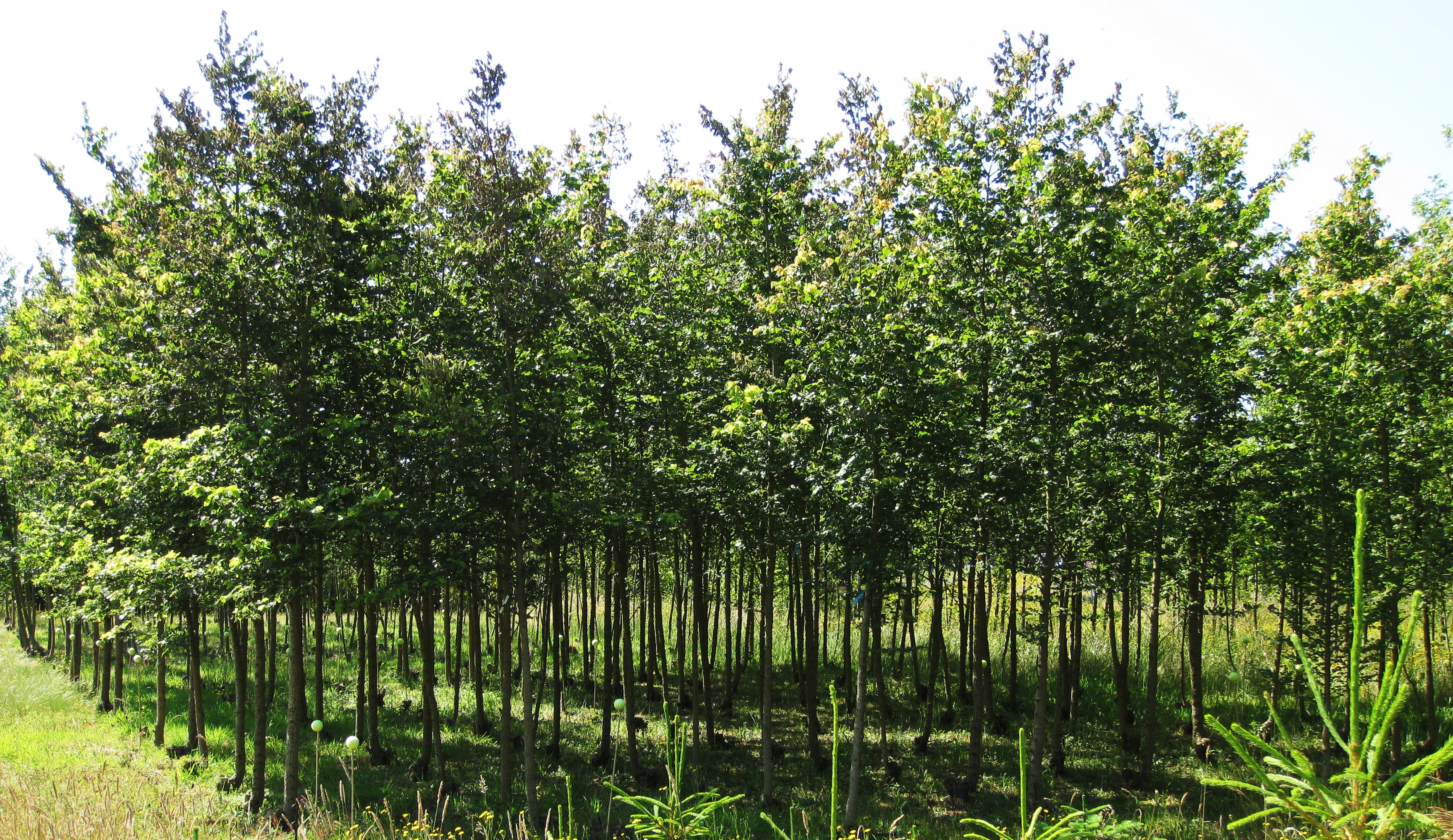

Supplement: Supplementary file 1 [file jof-07-00452-s001.zip › jof-1228785-SI/Supplementary files/Fig. S1 English elm.jpg]

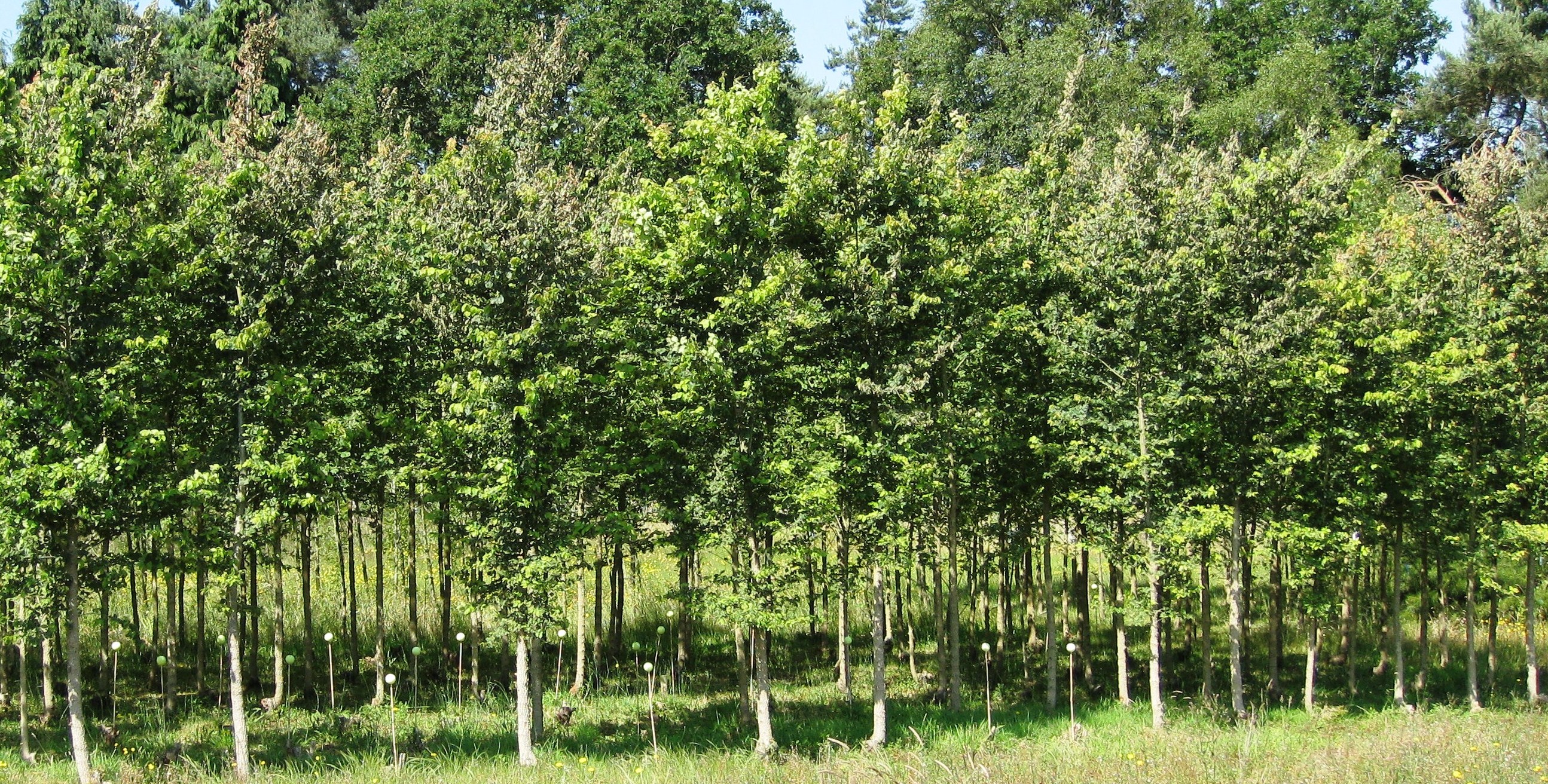

Supplement: Supplementary file 1 [file jof-07-00452-s001.zip › jof-1228785-SI/Supplementary files/Fig. S2 English elm.jpg]

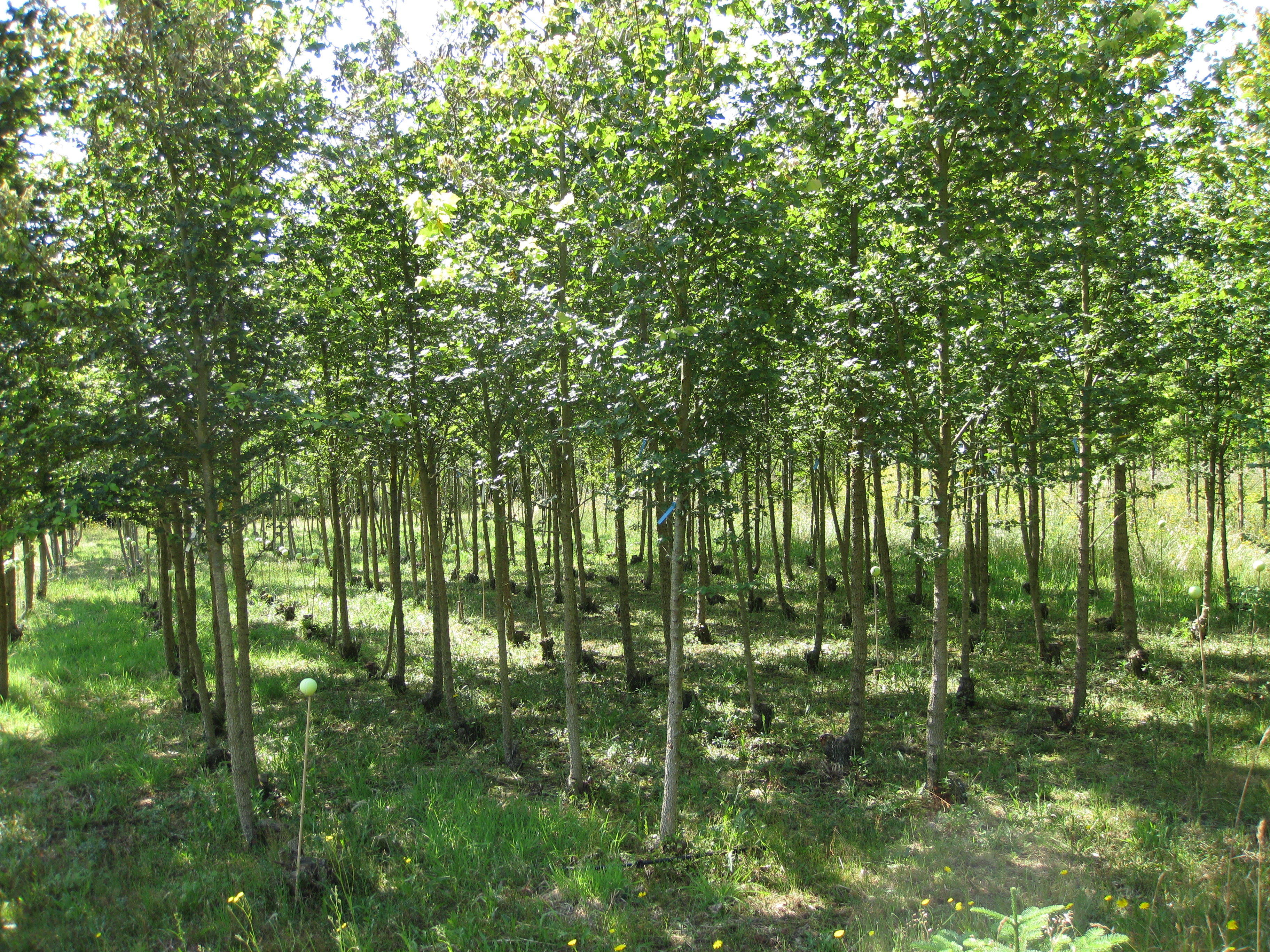

Supplement: Supplementary file 1 [file jof-07-00452-s001.zip › jof-1228785-SI/Supplementary files/Fig. S3 Commelin elm.jpg]

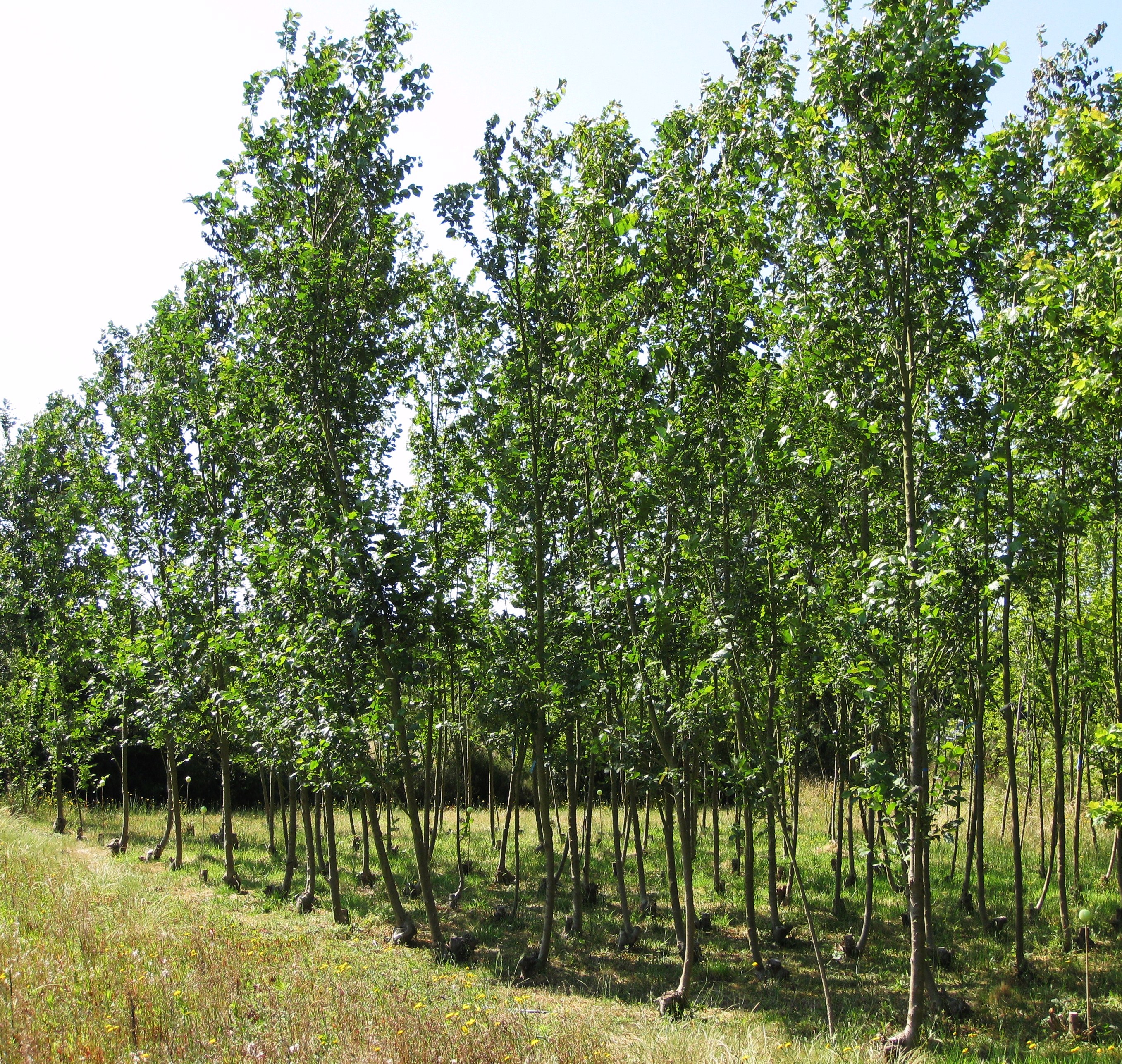

Supplement: Supplementary file 1 [file jof-07-00452-s001.zip › jof-1228785-SI/Supplementary files/Fig. S4 Commelin elm.jpg]
